# Leptin-induced autophagy regulates the immunomodulatory potential of adipose-derived mesenchymal stem cells through down regulating the expression of TSG-6

**DOI:** 10.1038/s41598-026-57830-6

**Published:** 2026-06-15

**Authors:** Lingling Xu, Liyan Ma, Lijun Liang, Xinyou Yu, Lan Zhang, Yuexuan Wu, Rui Ma, Jinhai Ma

**Affiliations:** 1 Department of Pediatrics, General Hospital of Ningxia Medical University, Yinchuan, 750000 Ningxia China; 2 Prenatal Diagnosis Center, General Hospital of Ningxia Medical University, Yinchuan, 750000 Ningxia China; 3 Department of Obstetrics and Gynecology, Peking University First Hospital Ningxia Women and Children’s Hospital (Ningxia Hui Autonomous Region Maternal and Child Health Hospital), Yinchuan, 750000 Ningxia China

**Keywords:** Obesity, Leptin, Autophagy, Adipose-derived mesenchymal stem cells, TSG-6, Immunomodulation, Cell biology, Diseases, Immunology, Stem cells

## Abstract

Adipose-derived mesenchymal stem cells (ADMSCs) possess modulatory functions in adipose tissue, which could help combat the development of obesity. It has been reported that the functions of ADMSCs are impaired by obese microenvironment. However, the mechanisms are not clearly understood. The study investigates the role of leptin on immunoinhibitory functions of ADMSCs in obesity, and primarily explored its possible mechanisms. ADMSCs were isolated and identified by morphological observation, flow cytometry and differentiation potential. The lipopolysaccharides (LPS)-stimulated macrophages were co-cultured with ADMSCs pretreated with or without leptin. The expression of macrophage-associated markers was detected by flow cytometry. The secretion of inflammatory cytokines was evaluated by ELISA. Autophagy and MAPK signaling related proteins were detected by western blotting. Furthermore, the expression of tumor necrosis factor-alpha-stimulated protein 6 (TSG-6) was detected by western blotting, qRT-PCR, and ELISA. ADMSCs belonged to CD73^+^ CD90^+^ CD105^+^/ CD14^-^ CD34^-^ CD45^-^ HLA-DR^-^ cells, and capable differentiation to adipogenic, osteogenic and chondrogenic cells. The immunosuppressive effect of ADMSCs on macrophage activation were restrained by leptin mediated the expression of TSG-6. Inhibition of leptin induced autophagy by Atg5 knockdown increased the expression of TSG-6, and promoted ADMSCs immunosuppression for macrophage activation. Furthermore, leptin induced autophagy and decreased the expression of TSG-6 in ADMSCs was dependent on the activation of p38 MAPK pathway. In conclusion, our study confirmed that leptin-induced autophagy regulated the immunomodulatory potential of ADMSCs through downregulating the expression of TSG-6 via the activation of p38 pathway. These findings reveal a novel mechanism and provide a potential molecular target for understanding the immunomodulatory dysfunction of ADMSCs in obesity.

**Supplementary Information:**

The online version contains supplementary material available at https://doi.org/10.1038/s41598-026-57830-6.

## Introduction

Globally, the prevalence of obesity has increased significantly in the last 2–3 decades^1^. Obesity is a major public health problem, contributing to the increase in the global burden of chronic diseases including mental health problems, diabetes, cardiovascular disease, and some types of cancers^2^.The association between obesity and the immune system has been described previously. Obesity can cause dysregulation of the immune system, affecting the balance and levels of cytokines, adipokines, and innate and adaptive immune cells, promoting the chronic low-grade inflammation^3,4^. Previous studies have shown that the chronic low-grade inflammation occurred in obesity represents a main factor in the pathogenesis of several chronic diseases^5^.

Adipose-derived mesenchymal stem cells (ADMSCs) are multipotent cells present in the adipose tissue. ADMSCs possess the characteristics of easy access, rapid proliferation in vitro and autologous transplantation^6^, and show great potential in the study of inflammatory diseases including obesity. ADMSCs are regarded as cardinal regulators for immune response in adipose tissues. They can modulate immune system cells’ proliferation and activation through direct cell–cell interaction and secretion of numerous cytokines and chemokines^7^. Intriguingly, obesity has been reported to alter ADMSCs biology, immune phenotype and functions^8^. Under the obese microenvironment, ADMSCs lose their beneficial functionalities, contributing to the development of obesity^7^. Thus, restoration of functional ADMSCs may provide a theoretical basis for alleviating obesity-related inflammation and related disorders. Understanding the mechanisms how obese microenvironment affects ADMSCs biology would help elucidate the immunoregulatory mechanism of ADMSCs.

Adipokines secreted by adipocytes play an important role in obese microenvironment^9^. Leptin is one of the best-studied hormones in energy homeostasis, with the abilities to inhibit adipogenesis, promote fat demarcation, increase insulin sensitivity, and promote a chronic inflammatory state^10^. Previous studies have revealed that leptin regulates the crosstalk between adipocytes and the immune system^11,12^. Wang et al.^13^ found that leptin promoted M1 macrophage polarization by activating the JNK/STAT3/AKT signaling pathways. Similarly, Monteiro et al.^14^ reported that leptin regulated macrophage fitness and contributed to obesity-induced inflammation and insulin resistance.

Previous studies have shown that leptin exerts its pro-inflammatory effects in obesity via inducing autophagy, an essential stress-response and homeostatic process^15^. It has been reported that MSCs characteristics including proliferation, differentiation, immunomodulation, were controlled by autophagy^16^. Tumor necrosis factor (TNF)-alpha-stimulated protein 6 (TSG-6) is key factor released from ADMSCs, which possesses the abilities to regulate autophagy and influence ADMSCs immunomodulatory properties^17,18^. On these bases, we hypothesized that leptin might contribute to obesity-induced inflammation by regulating autophagy and the autophagic and the expression of immunomodulatory factor TSG-6 in ADMSCs.

In the present study, we investigated how leptin-induced autophagy regulates​ the immunosuppressive potential of ADMSCs via modulating​ TSG-6 expression. We further evaluated effect of autophagy inhibition by genetic approaches in ADMSCs for the regualtion of macrophage polarization. Mechanistically, leptin was found to regulate autophagy and TSG-6 degradation via the p38 MAPK signaling pathway. The findings of this study provide novel mechanistic insights into the immunomodulatory disorder of ADMSCs in obesity.

## Methods

### Isolation and culture of ADMSCs

All experimental procedures involving human adipose tissue samples were performed in strict accordance with the principles outlined in the Declaration of Helsinki and relevant domestic institutional ethical regulations for human biomedical research. The collection of human subcutaneous adipose tissue and the whole research protocol of this study were reviewed and formally approved by the Ethics Committee of General Hospital of Ningxia Medical University (Approval NO. KYLL-2021-615). Written informed consent was obtained from all pediatric participants’ legal guardians prior to adipose tissue collection, and all personal identifiable information of donors was fully anonymized to protect patient privacy.

ADMSCs were isolated from adipose tissue samples according to the previous report^19^. The adipose tissue was minced using scissors and digested with 100 U/mL collagenase A and 10 μg/ mL DNase I at 37 °C for 2 h. After centrifugation of the suspension, the pellet was resuspended in in Nuwacell ncMission hMSC medium (Nuwacell, China) supplemented with 1 × GlutaMAX™ and 50 μg/mL gentamicin (TTermo Fisher Scientiffc, Carlsbad, California, USA). Replacement of the medium of the cultured cells and removal of the non-adherent cells were carried out after 48 h. ADMSCs were detached using trypsin–EDTA when they reached 80–90% confluence.

### Identification of ADMSCs

Surface phenotype analysis of ADMSCs was evaluated by flow cytometry. Briefly, ADMSCs were digested into individual cells for identification by flow cytometry. Cell aliquots (1 × 10^6^ cells/mL) were incubated with conjugated monoclonal antibodies used for flow cytometry against CD73-FITC, CD90-FITC, CD105-PE, CD14-PE, CD34-FITC, CD45-FITC, HLA-DR-FITC or conjugated isotypic controls (all from BD Biosciences, USA) at 4 ℃ for 30 min in the dark. Analysis was performed using FACS Celesta™ flow cytometer (BD Biosciences, San Diego, CA, USA).

To assess the adipogenic and osteogenic differentiation potential of ADMSCs, cells were plated into 24-well plates at 30,000 cells/well and cultured in adipogenic and osteogenic induction medium (OriCell, Guangzhou, China) at 37 °C and 5% CO_2_, respectively. After 21 days, adipogenesis or osteogenesis was identified by Oil Red O solution and Alizarin Red S, respectively. For chondrogenic differentiation, 1 × 10^6^ ADMSCs were resuspended in culture medium and centrifuged at 800 rpm for 5 min. Then, the pelleted cells were incubated with chondrogenic differentiation media (OriCell) for 21 days. Chondrogenic cells were determined by Alcian blue staining. All images were viewed on an Olympus microscope.

### Treatments

Human monocytic THP-1 cells were obtained from the Cell Bank of the Chinese Academy of Sciences. THP-1 cells were maintained in culture in RPMI 1640 culture medium containing 10% of fetal bovine serum (Gibco). THP-1 cells were co-cultured with 200 ng/mL phorbol 12-myristate 13-acetate (PMA, Sigma) to differentiate into macrophages. Recombinant leptin was obtained from MedChemExpress (MCE, USA). ADMSCs were pretreated with or without 250 ng/mL of leptin for 24 h. For the co-culture system, ADMSCs (10^5^/well) or leptin-stimulated ADMSCs were cultured in the upper chamber of a Transwell plate, and macrophages were grown in the lower chamber and stimulated with 100 ng/mL lipopolysaccharide (LPS, Sigma) for 48h^20^. The coculture system was added with 50 ng/mL recombinant human TSG-6 (R&D Systems, USA) and 5 μg/ mL TSG-6 neutralization antibody^21^ (Santa Cruz Biotechnology, Dallas, TX, USA) to verify the immunosuppressive function of TSG-6 derived from ADMSCs on macrophage M1 polarization. SB203580 is a p38 MAPK inhibitor. Chloroquine (CQ) is the inhibitor of autophagy. For the combined treatment of leptin and SB203580 (10 μM, HY-10256, MCE)/CQ (20 μM, HY-17589A, MCE), ADMSCs were pre-treated with SB203580/CQ for 6 h, followed by stimulation with leptin.

### Transmission electron microscopy (TEM)

Transmission electron microscopy (TEM) is often utilized to observe autophagosome or autolysosome, is critical to determine the autophagy formation. According to the protocol, ADMSCs stimulated with or without leptin washed in ice-cold PBS, fixed with 2% glutaraldehyde (Sigma-Aldrich, G5882), rinsed twice with PBS, and then post-fixed in 1% osmium tetroxide (Sigma-Aldrich, 75,632), dehydrated, treated with propylene oxide (Sigma Aldrich, 82,320), and embedded in epoxy resin (Sigma Aldrich, A3183). Subsequently, ultrathin sections ranging from 60 to 90 nm were prepared using an ultramicrotome, then stained with lead citrate (Sigma-Aldrich, 15,326) and images were acquired using a transmission electron microscope (JEM-1400FLASH, JEOL, Japan).

### mRFP-GFP-LC3 adenovirus double label assay

ADMSCs were transfected with mRFP‐GFP‐LC3 (Hanbio, Shanghai, China) and cultured for 24 h. Then, the cells were treated with leptin or negative control for another 8 h. Subsequently, the cells were fixed with paraformaldehyde (PFA) and nuclei were stained with DAPI (Sigma). Finally, the images of cells were taken using fluorescence microscopy.

### Lentivirus construction and infection

The specific Atg5 shRNA lentiviruses and scrambled control (sh-NC) were designed and synthesized by GenePharma Co., Ltd. The target nucleotide sequences were as follows: Atg5-shRNA, 5′—GAAGTTTGTCCTTCTGCTA-3′ (sh-Atg5); scrambled control-shRNA, 5′—CCTAAGGTTAAGTCGCCCTCG-3′ (sh-NC). A multiplicity of infection of 50 of lentiviruses was utilized for ADMSCs infection.

### Flow cytometry

THP-1 cells after different treatments were first harvested and fixed overnight in 1% paraformaldehyde (PFA) at 4 °C. Staining was performed using antibodies against CD86-FITC (BD Biosciences) at 4 ℃ for 30 min in the dark. All samples were pre-incubated with Fc-block (BioLegend) prior to staining. Analysis was performed using FACS Celesta ffow cytometer (BD Biosciences, San Diego, CA, USA).

### Real-time quantitative polymerase chain reaction (qRT-PCR)

Total RNA within cells was extracted by cell lysis using TRIZOL reagent and reversely transcribed into cDNA by cDNA Synthesis Kit (TaKaRa Bio, USA). qRT-PCR experiments were performed using TB Green ® Premix Ex Taq ™ II (Tli RNaseH Plus), Bulk (Takara, Tokyo, Japan) kits. The PCR procedure was set up according to the instructions. Data processing was performed using the 2^-ΔΔCt^ method. GAPDH gene was used as a control^22^. Primer information was as follows: TSG-6: forward 5’- GGCTGGCAGATACAAGCTCA -3’, reverse 5’-TCAAATTCACATACGGCCTTGG—3’. GAPDH: forward 5’- AACATCATCCCTGCTTCCAC—3’, reverse 5’-GACCACCTGGTCCTCAGTGT-3’.

### Enzyme-linked immunosorbent assay (ELISA)

Cell culture supernatants were collected after different treatments. The protein levels of TSG-6, TNF-α, IL-6, in the cell culture supernatants were detected by human TSG-6 ELISA kit (Shanghai Macklin Biochemical Technology, China), human TNF-α ELISA kit (#PT518, Beyotime Biotech, China), human IL-6 ELISA kit (#PI330, Beyotime), respectively, according to the manufacturer’s instruction.

### Western blot analysis

Protein samples from ADMSCs or transfected cells with different treatments were extracted using Minute Total Protein Extraction Kit (Invent Biotechnologies, USA). The content of each sample was determined by BCA protein assay kit (Beyotime). Subsequently, the proteins were separated and transferred to PVDF membranes (Millipore, USA). Following by blocking with 5% skimmed milk, the membranes were incubated with primary antibodies specific to TSG-6 (1:1000, ab267469, Abcam, USA), microtubule-associated protein 1 light chain 3 [LC3, 1:1000, #12,741, Cell Signaling Technology (CST), Danvers, USA], p62 (1:1000, #39,749, CST), Atg5 (1:1000, #12,994, CST), phosphorylated (p)-p38 (1:1000, #4511, CST), p38 (1:1000, #9212, CST), extracellular regulated kinase 1/2 (ERK1/2, 1:1000, #9102, CST), p-ERK1/2 (1:1000, #4370, CST), c-Jun N-terminal kinase (JNK, 1:1000, #9252, CST), p-JNK (1:1000, #4668, CST), and β-tubulin (1:1000, #2128, CST). Then, the membranes were incubated with HRP-linked goat anti-rabbit IgG secondary antibodies (1:3000, # 7074, CST). ECL (Millipore) solution was used to observe the protein band. Relative protein expression was quantified by BioImaging Systems.

### Co-immunoprecipitation (Co-IP) assay

Processed ADMSCs were collected and prepared according to the manufacturer’s instructions for the Co-IP kit (Thermo Scientiffc™ Pierce™, USA). Briefly, The Pierce Protein A/G magnetic beads were prewashed twice with 2 mL of 1 × modiffed coupling buffer. The beads were gently mixed and incubated with 100 µL of either anti-p62 antibody solution or negative control IgG solution. Then the beads washed by 1 × modified crosslinking buffer were combined with the DSS crosslinker. The beads were washed with elution buffer and the immunoprecipitation lysis/wash buffer in turn. The antibody-crosslinked beads were washed by the immunoprecipitation lysis/wash buffer before incubated overnight with the cell lysate at 4 °C, followed by a final wash in pure water. Subsequently, the bound antigen was eluted and analyzed following the previously described Western blotting method.

### Justification of leptin concentration

The concentration of leptin (250 ng/mL) used in this study was selected based on its relevance to the pathological microenvironment of obesity. Several studies have reported that leptin levels in the interstitial fluid of adipose tissue from obese individuals can range from 20 to over 500 ng/mL, with severe obesity often associated with levels in the hundreds of ng/mL^23,24^. Specifically, a study by Considine, R. V et al. demonstrated that leptin concentrations in the adipose tissue of morbidly obese patients reached approximately 200–300 ng/mL^23^. Therefore, the dose of 250 ng/mL employed herein is representative of the pathophysiological leptin levels encountered in obese adipose tissue and is widely used in vitro to mimic the obese state^25^.

### Statistical analysis

Data were analyzed using SPSS software (version 23.0) and are presented as mean ± standard deviation (SD). All experiments were independently repeated at least three times (n ≥ 3), and the specific sample size for each experiment is provided in the corresponding figure legends. The normality of data distribution was assessed using the Shapiro–Wilk test, and the homogeneity of variances was verified using Levene’s test. Based on these assessments, parametric tests were deemed appropriate. For comparisons between two groups, an unpaired two-tailed Student’s t-test was used. For comparisons among more than two groups, one-way analysis of variance (ANOVA) was performed, followed by the Least Significant Difference (LSD) post hoc test for multiple comparisons. *P* < 0.05 was considered statistically significant.

## Results

### Characterization of ADMSCs cultured in serum-free medium

**Fig. 1:**
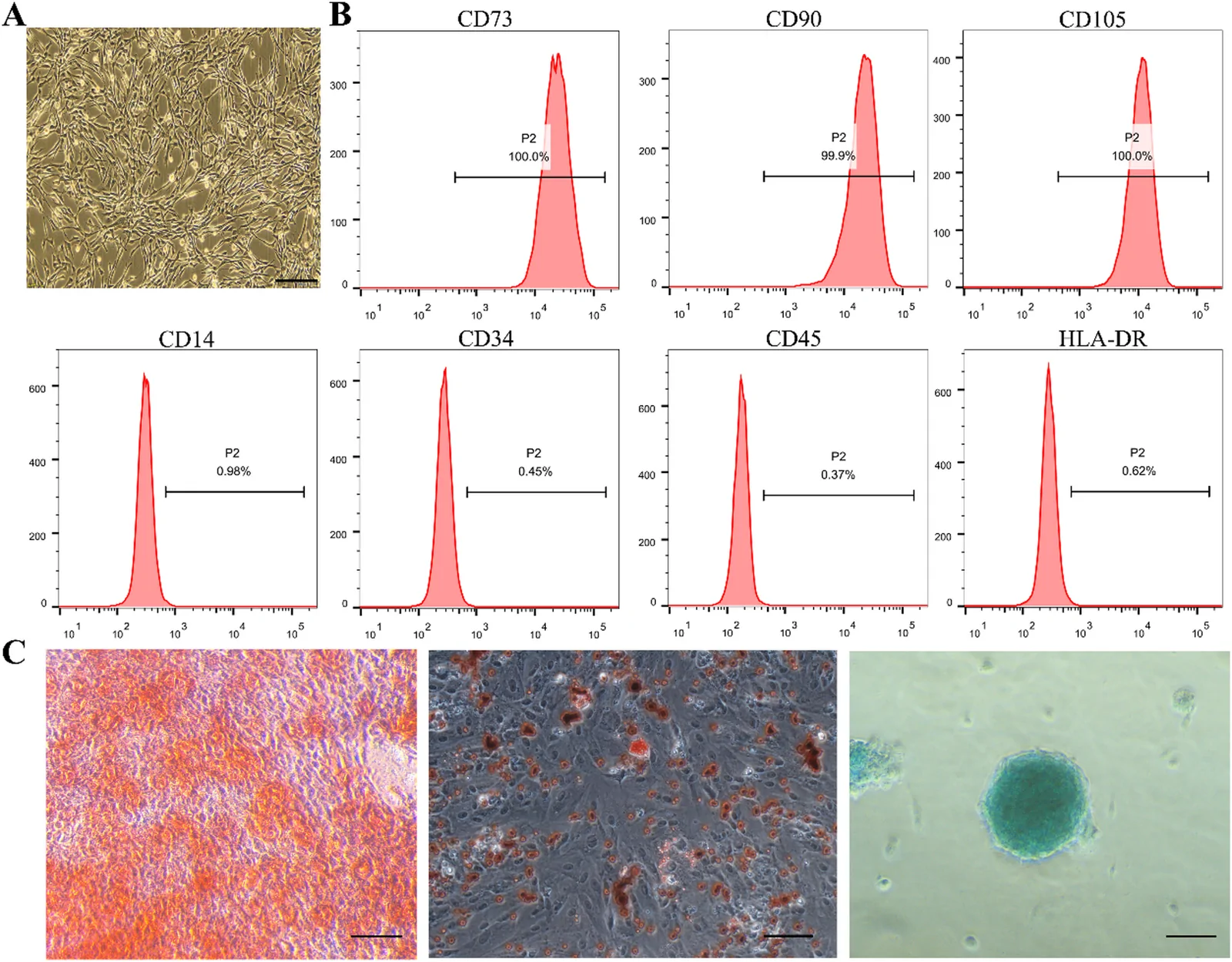
Characterization of isolated adipose-derived mesenchymal stem cells (ADMSCs) in serum-free medium. (**A**) The morphology of ADMSCs exhibited the typical spindle shape of MSCs. (**B**) ADMSCs were highly positive for mesenchymal stem cells surface markers, including CD73, CD90, and CD105, but negative for CD14, CD34, CD45 and HLA-DR by flow cytometry analysis. (**C**) The multipotency of ADMSCs was detected via osteogenic, adipogenic, and chondrogenic differentiation using alizarin red staining, oil red O staining and alcian blue staining. Data are presented as mean ± SD (n = 3 independent experiments). Bars in A and C: 100 µm.

The isolated ADMSCs demonstrated a fibroblast-like, spindle-shaped morphology (Fig. 1A). The flow cytometry analysis showed that ADMSCs were highly positive for CD73, CD90, and CD105, but negative for CD14, CD34, CD45 and HLA-DR (Fig. 1B). The multipotency of ADMSCs was detected via osteogenic, adipogenic and chondrogenic differentiation. Osteogenic differentiation showed the presence of calcium deposition, adipogenic differentiation exhibited lipid droplets in the cytoplasm, and chondrogenic differentiation showed typical glycosaminoglycans in cartilage (Fig. 1C).

### Leptin reduces the inhibitory effect of ADMSCs on macrophage polarization towards M1 phenotype

**Fig. 2:**
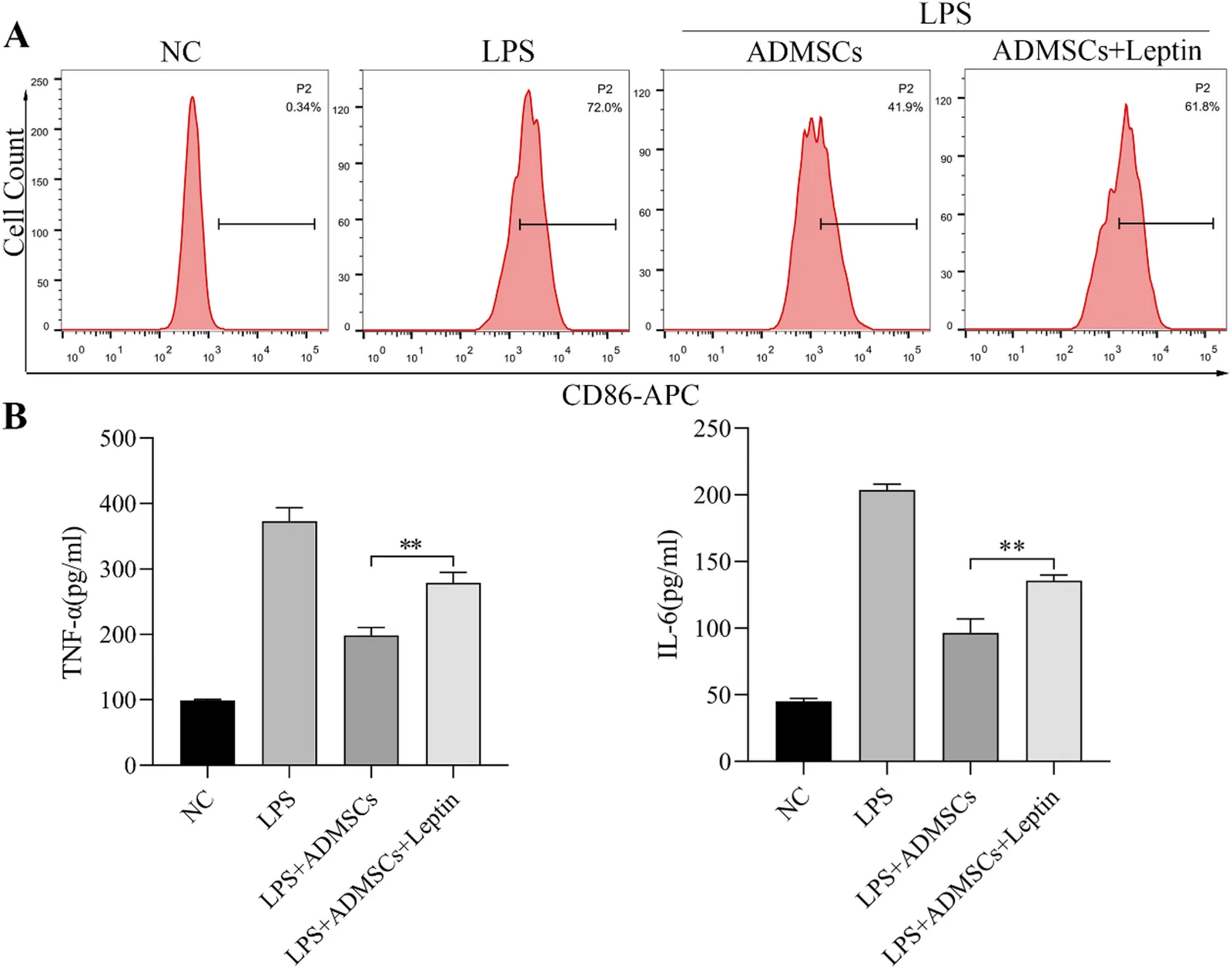
Leptin reduces the inhibitory effect of ADMSCs on macrophage polarization towards M1 phenotype. (**A**) Flow cytometry analysis of CD86 expression in macrophages. (**B**) The secretion levels of M1 pro-inflammatory cytokines TNF-α and IL-6 in cell culture supernatants were detected via ELISA assay. Data are presented as mean ± SD (n = 3 independent experiments). *P < 0.05, **P < 0.01.

The LPS-stimulated macrophages were co-cultured with ADMSCs (with or without leptin), and the macrophage-associated markers were detected by flow cytometry. The results indicated that ADMSCs significantly reduced the expression of CD86, a marker associated with the M1 phenotype (Fig. 2A). In contrast, this reduction was attenuated when macrophages were co-cultured with leptin-pretreated ADMSCs, which instead led to an increase in CD86 expressio (Fig. 2A). The detection of inflammatory cytokines further confirmed our findings. M1 macrophages secreted IL-6 and TNF-α^26,27^. In this study, the above cytokines were detected in cell culture medium using ELISA. Consistent with the flow cytometry data, macrophages co-cultured with untreated ADMSCs secreted lower levels of IL-6 and TNF-α compared to the LPS-stimulated control group (Fig. 2B). However, leptin pretreatment of ADMSCs attenuated this suppressive effect, leading to higher secretion of both cytokines compared to the co-culture with untreated ADMSCs (Fig. 2B).

### Leptin downregulated the expression of TSG-6 in ADMSCs

**Fig. 3:**
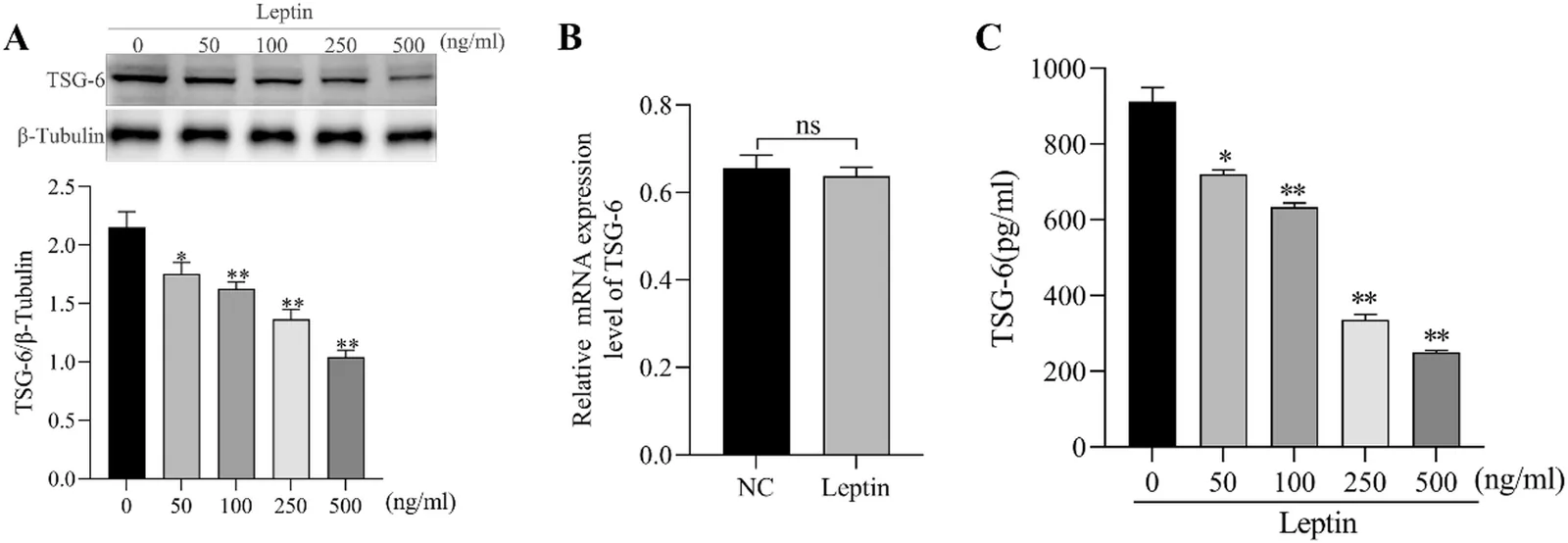
Leptin downregulated the expression of TSG-6 in ADMSCs. (**A**) ADMSCs were treated with different concentrations of leptin (0, 50, 100, 250 and 500 ng/mL), and the expressions of TSG-6 were detected by western blot analysis. (**B**) The effect of leptin on the expression of TSG-6 mRNA was determined by RT-PCR. (**C**) The effect of leptin on the secretion of TSG-6 in cell culture medium was detected by ELISA. Data are presented as mean ± SD (n = 3 independent experiments) *P < 0.05, **P < 0.01.

The effect of leptin on TSG-6 expression in ADMSCs was evaluated at both the protein and transcriptional levels. First, ADMSCs were treated with increasing concentrations of leptin (0, 50, 100, 250, and 500 ng/mL). Western blot analysis demonstrated a dose-dependent decrease in TSG-6 protein levels following leptin treatment (Fig. 3A). To determine whether this reduction occurred at the transcriptional level, TSG-6 mRNA expression was analyzed by qRT-PCR. In contrast to the protein data, leptin treatment did not significantly alter TSG-6 mRNA levels compared to the untreated control (Fig. 3B). Finally, the secretion of TSG-6 into the cell culture medium was measured by ELISA. Consistent with the intracellular protein findings, leptin treatment led to a significant reduction in the level of secreted TSG-6 protein (Fig. 3C).

### Leptin induced autophagy in ADMSCs

**Fig. 4:**
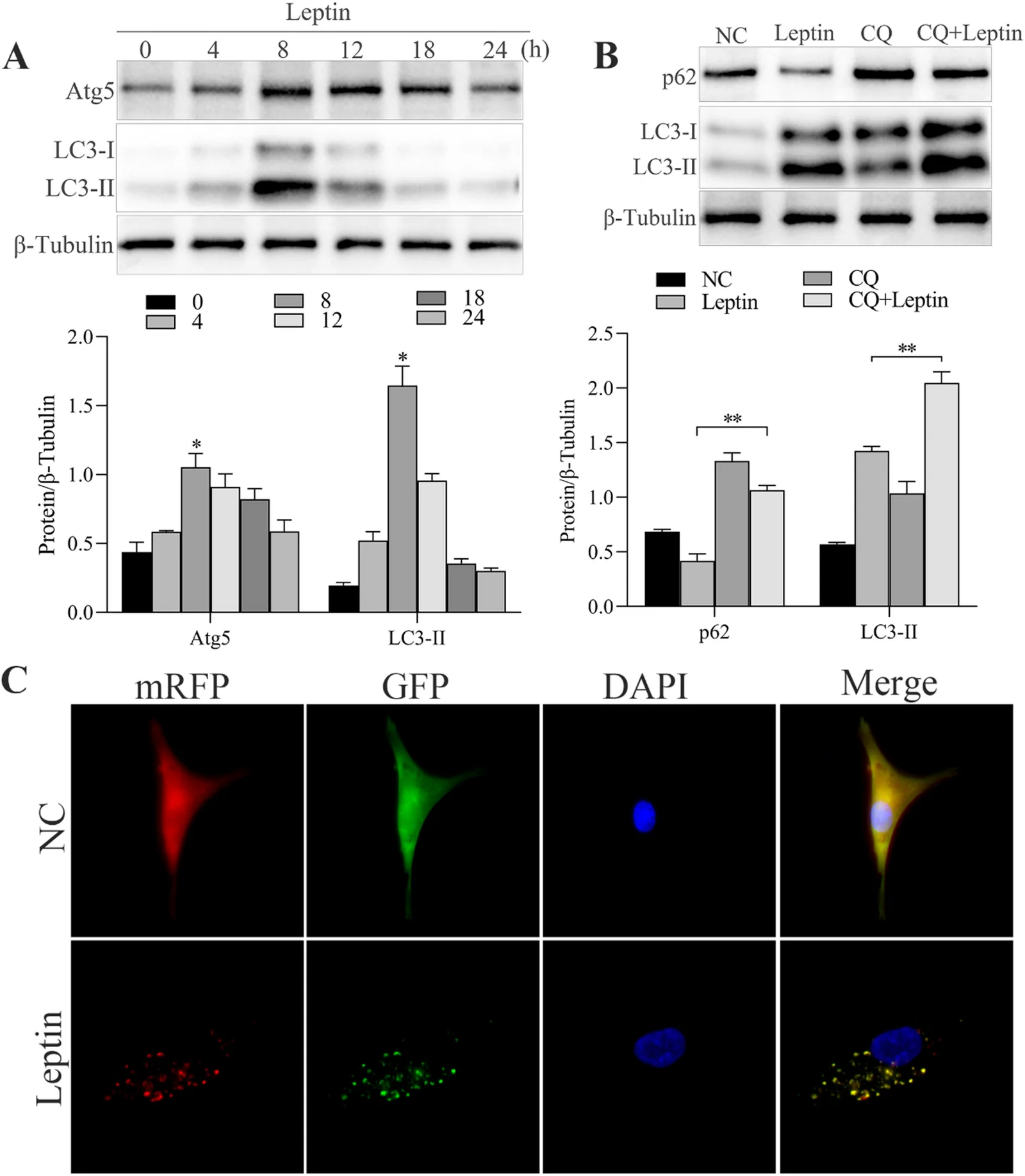
Leptin induced autophagy in ADMSCs. (**A**) ADMSCs were treated with leptin at various periods of time, and the expression of LC3-II was detected by western blot at the indicated times. (**B**) ADMSCs were pretreated with chloroquine (CQ) and stimulated with leptin, the expressions of p62 and LC3-II were evaluated by western blot analysis. (**C**) ADMSCs stimulated with or without leptin, the process of autophagic flux was observed by mRFP-GFP-LC3 adenovirus double label assay. Data are presented as mean ± SD (n = 3 independent experiments). *P < 0.05, **P < 0.01.

To assess whether leptin induces autophagy in ADMSCs, cells were treated with leptin and harvested at indicated time points (0, 4, 8, 12, 18, and 24 h). Western blot analysis showed that leptin treatment significantly increased the levels of the autophagy marker LC3-II over time, with the most pronounced increase occurring at 8 h (Fig. 4A). To differentiate between autophagosome accumulation and enhanced autophagic flux, ADMSCs were co-treated with leptin and chloroquine (CQ), a lysosomal inhibitor that blocks autophagic flux. Compared to leptin treatment alone, the combination of leptin and CQ resulted in a further accumulation of LC3-II (Fig. 4B). Concurrently, leptin treatment reduced the levels of p62, a substrate degraded by autophagy (Fig. 4B). Furthermore, autophagosome formation was visualized using the mRFP-GFP-LC3 adenovirus double-label assay. Fluorescence microscopy revealed a greater number of cytoplasmic autophagosomes (yellow puncta, representing autophagosomes) in leptin-treated ADMSCs compared to untreated controls (Fig. 4C).

### Leptin-induced autophagy inhibited the expression of TSG-6

**Fig. 5:**
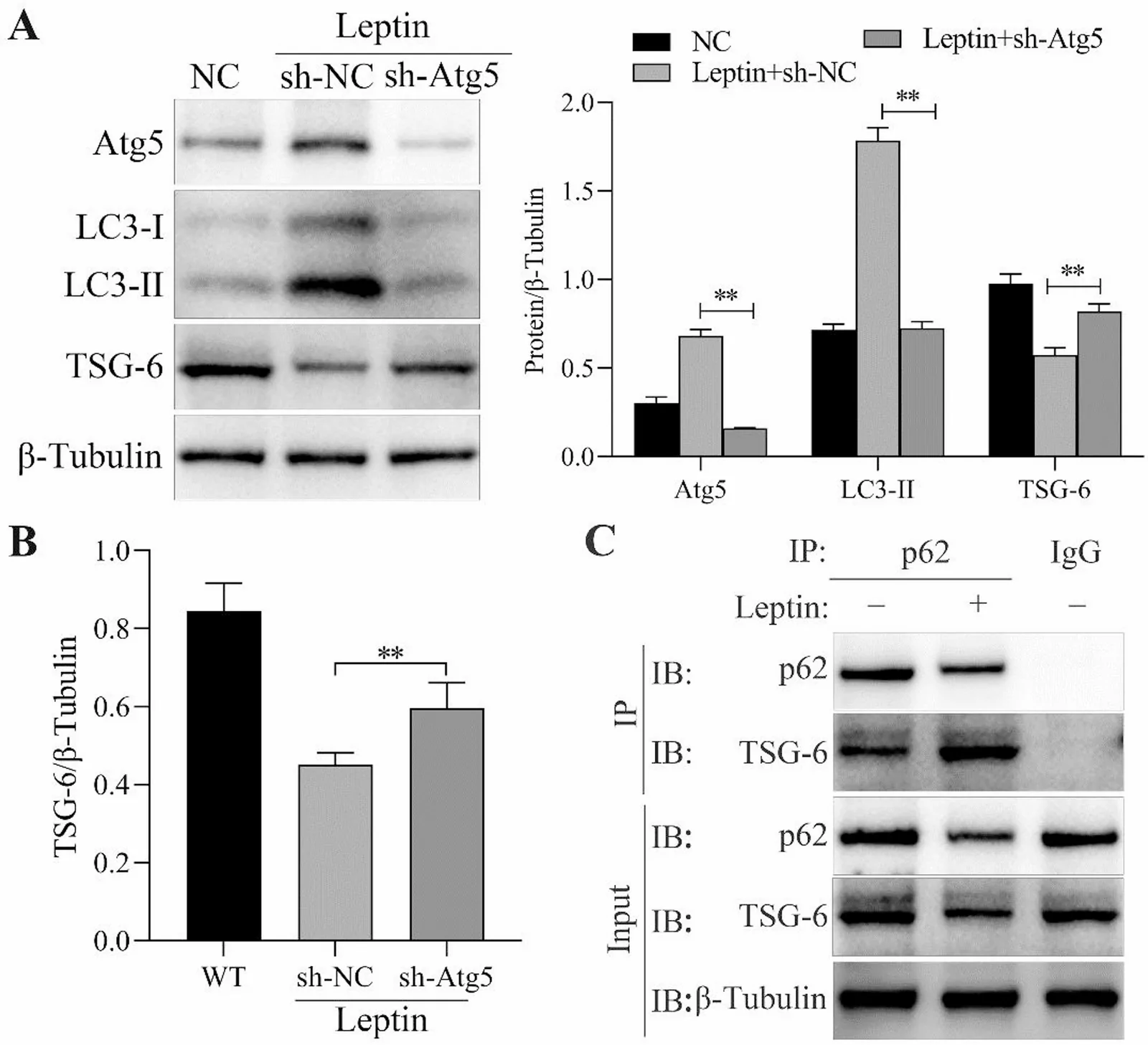
Leptin-induced autophagy inhibited the expression of TSG-6. ADMSCs were infected with control lentivirus (sh-NC) or lentivirus expressing shRNA targeting Atg5 (sh-Atg5), and treated with or without leptin. The expressions of Atg5, LC3-II and TSG-6 were analyzed by western blot analysis (**A**). ADMSCs were infected with sh-NC and sh-Atg5 lentivirus and treated with leptin, the expression of TSG-6 protein was determined by ELISA (**B**). The interaction between p62 and TSG-6 in Leptin-treated ADMSCs was examined via a Co-IP assay (**C**). Data are presented as mean ± SD (n = 3 independent experiments). *P < 0.05, **P < 0.01.

To determine the role of autophagy in leptin-mediated TSG-6 downregulation, ADMSCs were transduced with lentivirus expressing shRNA targeting Atg5(sh-Atg5) or a control shRNA (sh-NC), followed by leptin treatment. As expected, knockdown of Atg5effectively reduced the protein levels of both Atg5 and the autophagy marker LC3-II in leptin-treated ADMSCs (Fig. 5A).The impact of autophagy inhibition on TSG-6 was then assessed. Western blot and ELISA analyses revealed that, in the presence of leptin, sh-Atg5 transduction led to a significant increase in both intracellular and secreted TSG-6 protein levels compared to the sh-NC control (Fig. 5A and B).To investigate the mechanism underlying autophagic degradation of TSG-6, co-immunoprecipitation (Co-IP) was performed to examine the potential interaction between TSG-6 and the selective autophagy adapter protein p62. An interaction between p62 and TSG-6 was clearly detected in leptin-treated ADMSCs (Fig. 5C).

### Autophagy inhibition enhances immunosuppressive effect of ADMSCs under leptin exposure by increasing TSG-6 expression

**Fig. 6:**
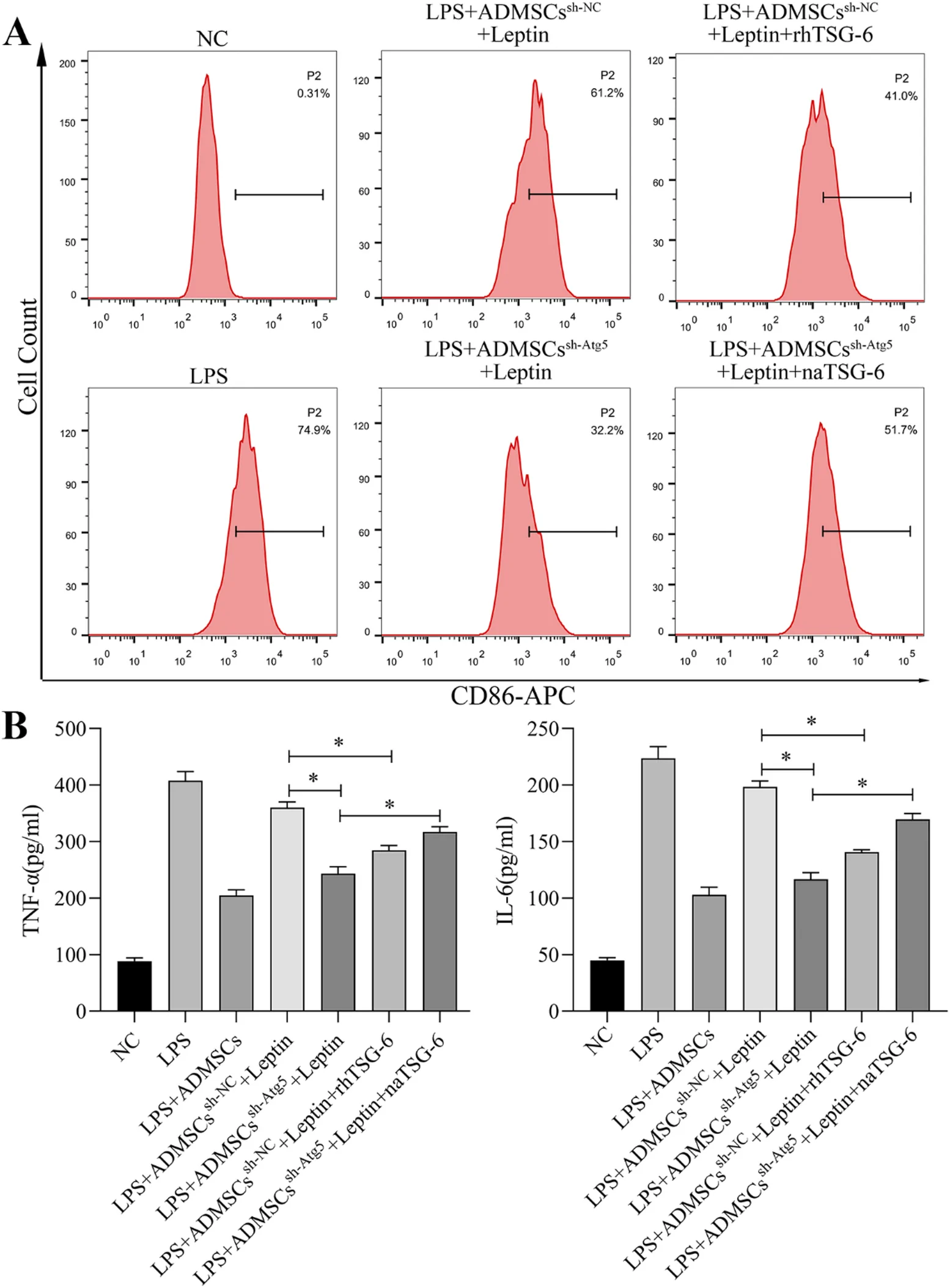
Autophagy inhibition alleviated leptin’s effects on macrophage polarization. Leptin-stimulated ADMSCs transfected with sh-Atg5 or sh-NC were co-cultured with LPS-stimulated macrophages. The macrophage-associated markers were detected by flow cytometry (**A**), and the macrophage-associated cytokines were detected by ELISA (**B**). Data are presented as mean ± SD (n = 3 independent experiments). *P < 0.05, **P < 0.01.

To further determine whether the immunomodulatory functions of ADMSCs was influenced by leptin induced autophagic degradation of TSG-6, leptin-stimulated ADMSCs transfected with sh-Atg5 or sh-NC were co-cultured with LPS-stimulated macrophages. The results from flow cytometry analysis showed that macrophages co-cultured with leptin-stimulated sh-Atg5-ADMSCs expressed lower levels of CD86 than those co-cultured with leptin-stimulated sh-NC-ADMSCs (Fig. 6A). To further determine the immunomodulatory function of TSG-6, 50 ng/mL recombinant human TSG-6 and 5 μg/mL TSG-6 neutralization antibody were added to the co-culture system to increase or neutralize TSG-6 levels. The results demonstrated that recombinant human TSG-6 observably decreased CD86 positive macrophages ratio compared to the sh-NC infection group, while TSG-6 neutralization antibody reversed this effect in the sh-Atg5 infection group (Fig. 6A). Moreover, macrophages co-cultured with leptin-stimulated ADMSCs^sh-Atg5^ produced dramatically lower levels of IL-6 and TNF-α, comparing to ADMSCs^sh-NC^ group, while recombinant human TSG-6 and TSG-6 neutralization reversed the expression tendency of IL-6 and TNF-α respectively (Fig. 6B).

### Leptin downregulated the expression of TSG-6 in ADMSCs by autophagy via the activation of p38 MAPK pathway

**Fig. 7:**
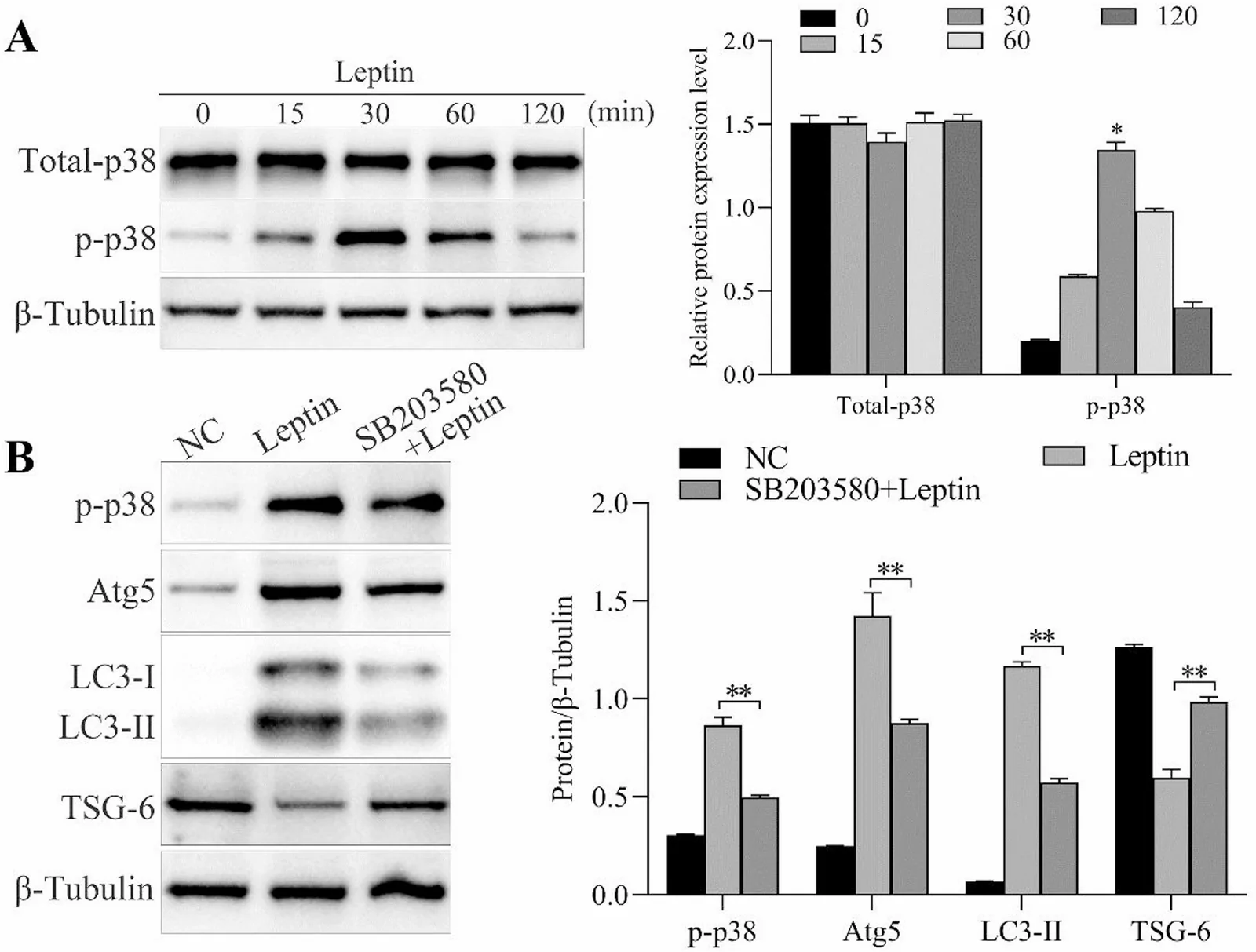
Leptin downregulated the expression of TSG-6 in ADMSCs by autophagy via the activation of p38 MAPK pathway. (**A**) ADMSCs were treated with leptin, and the expression of MAPK pathway proteins was detected by western blot. (**B**) ADMSCs were pretreated with p38 MAPK inhibitor SB203580 prior to leptin treatment, and the expressions of p-p38, Atg5, LC3-II, and TSG-6 were assessed by western blot analysis. Data are presented as mean ± SD (n = 3 independent experiments). *P < 0.05, **P < 0.01.

MAPK signaling pathway have been found to take part in the regulation of autophagy as well as leptin mediated biological response^26–31^. Thus, we focused on this pathway to clarify the mechanism of TSG-6 regulation by leptin in ADMSCs. Western blot analysis showed that the levels of p-p38 dynamically increased with exposure to leptin in ADMSCs (Fig. 7A). Using p38 MAPK inhibitor SB203580 to interfere p38 signaling, we found that SB203580 reversed the effects of leptin on TSG-6 and LC3-II expressions. Compared to the leptin group, the leptin + SB203580 group exhibited lower expression levels of p-p38, Atg5 and LC3-II, but a higher expression level of TSG-6 (Fig. 7B).

## Discussion

Obesity is major public health problem all over the world, contributing to the increase risk of several chronic diseases. ADMSCs possess modulatory functions in adipose tissue, which could help combat the development of obesity^7^. However, Inflammatory milieu and abnormal metabolites in obese tissue alter and reduce the functionality of ADMSCs^32^. Thus, ADMSCs in obesity are defective in various functionalities including immunomodulation. Restoring the function of ADMSCs in the pathological microenvironment is a potential treatment for obesity and its associated diseases^7^. In the present study, we found that immunosuppressive effect of ADMSCs on macrophage polarization towards M1 phenotype were restrained by leptin pretreatment. Mechanistically, leptin was found to induce autophagy of ADMSCs and downregulate the expression of TSG-6 in ADMSCs via the activation of p38 MAPK pathway. Our study demonstrated for the first time that the immunoinhibitory functions of ADMSCs in obese microenvironment was restricted by leptin-mediated autophagy, provide a mechanistic basis for understanding the immunomodulatory disorder of ADMSCs under obese conditions.

Macrophages are the most abundant infiltrated immune cells in obese adipose tissue^7^. Macrophages with a shift from the M2 to the M1 phenotype is one of the most important characteristics of obese microenvironment^7^. Several reports have addressed MSC-macrophage cross talk, by which proinflammatory M1 macrophage polarization could be suppressed^33^. In the present study, we also found that ADMSCs isolated from children’s subcutaneous adipose tissues could inhibit macrophages toward M1 polarization, confirming the immunomodulatory potential of ADMSCs. Leptin is a product of the obese (*ob*) gene secreted by white adipose tissue, which promotes a chronic inflammatory state in obese^12^. Previous studies have reported that enhancing leptin-related signaling could exacerbate obesity-related inflammation through inducing the activation of M1 macrophages^13,14^. Similarly, our study found that leptin restrained the immunosuppressive effect of ADMSCs on macrophage polarization. leptin pre-treatment alleviated the inhibitory role of ADMSCs for macrophage polarization towards M1 phenotype, with increased M1 markers CD86 positive cells ratio as well as secretion level of M1 macrophage-related cytokine TNF-α and IL-6. Our findings confirmed that the immunoinhibitory functions of ADMSCs in obese microenvironment was restricted by leptin. In addition, THP‑1‑derived macrophages represent a well‑established and widely accepted model for investigating macrophage polarization and inflammatory responses, and their responses are highly consistent with those of human primary macrophages^26,27^.

TSG-6 is an inflammation-associated protein, which could be secreted by ADMSCs^17^. Previously, TSG-6 has been demonstrated as a multifunctional protein with anti-inflammatory and tissue-protective properties^34^. Furthermore, this immunoregulatory protein is regarded as a pivotal mediator of the therapeutic efficacy of MSCs in diverse pathological conditions^35^. Song et al^17^. found that TSG-6 released from ADMSCs could induce a macrophage phenotypic switch from M1 to M2, thereby ameliorating inflammatory bowel disease. Jha et al.^36^ reported that ADMSCs responded to an inflammatory milieu by secreting higher levels of TSG-6. In this study, we found that leptin down-regulated the expression of TSG-6 in ADMSCs. Our study is the first study to reveal the relationship between leptin and TSG-6, suggesting that leptin restricted the immunoinhibitory functions of ADMSCs through down-regulating TSG-6 expression.

Autophagy is a conserved lysosomes degradation process, which is the key contributor to the therapeutic action of MSCs^37^. Leptin levels have been shown previously to be positively associated with the expression levels of autophagy genes including Atg5^38^. Several studies revealed that leptin exerted its pro-inflammatory effects in obesity via inducing autophagy^15,28,30^. Our study showed that leptin resulted in remarkable elevation of LC3-II in ADMSCs, accompanied with accumulation of autophagosomes. Using the CQ to arrest autophagy flux, LC3-II levels in ADMSCs treated with leptin were further up-regulated. Taken together, our findings confirmed that leptin in obese microenvironment had capability to trigger autophagy in ADMSCs.

Previous studies suggested that the expressions of immunosuppressive molecules in inflammatory microenvironment were regulated by autophagy induced by pathological milieu in MSCs^16,39^. To evaluate the reduction of TSG-6 in leptin-treated ADMSCs was triggered by autophagy, sh-Atg5 was used to inhibit the autophagy of ADMSCs. We found that the expression of TSG-6 in ADMSCs was reversed by Atg5 knockdown under leptin exposure. Furthermore, p62 as a selective autophagy adaptor take part in protein degradation and maintains proteostasis^40,41^. Our data verified that an interaction between the autophagy adaptor protein p62 and TSG-6 in ADMSCs treated with leptin. Critically, our study also revealed, under the treatment of leptin, autophagy inhibition promoted immunosuppressive function of ADMSCs. Thus, our study clarified that leptin-induced autophagy took part in the TSG-6 expression and the immunoinhibitory functions of ADMSCs in the obese microenvironment. However, it should be noted that ubiquitination of target proteins is typically required for p62-mediated selective autophagy. Whether TSG-6 is ubiquitinated during leptin-induced autophagic degradation needs to be further explored in future investigations.

MAPK is the necessary upstream signaling pathway to regulate inflammation^42^. It is well known that the molecules of the MAPK family involved in inflammation mainly include JNK, ERK1/2, and p38^43^. Data from previous studies demonstrated that leptin regulated some cellular functions through MAPK signaling^29,31,44^. In the present study, we found that the levels of p-p38 were dynamically increased with exposure to leptin in ADMSCs. p38 MAPK signaling has been reported to regulate the immune microenvironment and macrophage polarization^45^. In addition, p38 MAPK signaling is involved in the modulation of autophagy process^46^. Using p38 MAPK inhibitor SB203580 to interfere p38 signaling in this study, we found that the expression of the autophagic biomarkers were declined. Our study suggested that leptin-induced autophagy downregulated the expression of TSG-6 and regulated the immunomodulatory potential of ADMSCs via the activation of p38 MAPK pathway.

It is important to note that the majority of our mechanistic investigations were conducted using the THP-1 human monocytic cell line as a model for macrophages. While THP-1 cells are a well-established and widely used model for studying macrophage biology and polarization, and their responses often correlate with primary human macrophages^47^, validation in primary human macrophages would further strengthen the translational relevance​ of our findings. Future studies utilizing macrophages derived from human peripheral blood mononuclear cells (PBMCs) or adipose tissue-resident macrophages are warranted to confirm the translational potential of the leptin-autophagy-TSG-6 axis identified here. ore broadly, a primary limitation of this study is the lack of in vivovalidation. The therapeutic implications proposed herein, therefore, remain hypothetical and require confirmation in appropriate animal models of obesity. Future work should directly investigate whether modulating the leptin-autophagy-TSG-6 axis in ADMSCs in vivocan ameliorate obesity-associated inflammation and metabolic dysfunction.

## Conclusion

In conclusion, our study confirmed that leptin, an important proinflammatory marker mainly expressed in adipose tissue, restricted the immunoinhibitory functions of ADMSCs in obese microenvironment. The mechanism research revealed that leptin-induced autophagy regulated the immunomodulatory potential of ADMSCs through downregulating the expression of TSG-6 via the activation of p38 MAPK pathway. Our study demonstrated for the first time that the immunoinhibitory functions of ADMSCs in obese microenvironment was restricted by leptin-mediated autophagy. T Collectively, our findings elucidate a novel molecular mechanism by which leptin, via the p38 MAPK-autophagy axis, downregulates TSG-6 and impairs ADMSC immunomodulation. This mechanism warrants further investigation as a potential target for improving ADMSC function in metabolic disorders.

## Supplementary Information

Below is the link to the electronic supplementary material.


Supplementary Material 1



Supplementary Material 2



Supplementary Material 3



Supplementary Material 4



Supplementary Material 5


## Data Availability

The data that support the findings of this study are available from the corresponding author upon reasonable request.
