# Supplementary material for: Leptin-induced autophagy regulates the immunomodulatory potential of adipose-derived mesenchymal stem cells through down regulating the expression of TSG-6
Source: Sci Rep. 2026 Jun 15;16:27363. doi: 10.1038/s41598-026-57830-6 (PMC13530140; doi:10.1038/s41598-026-57830-6)

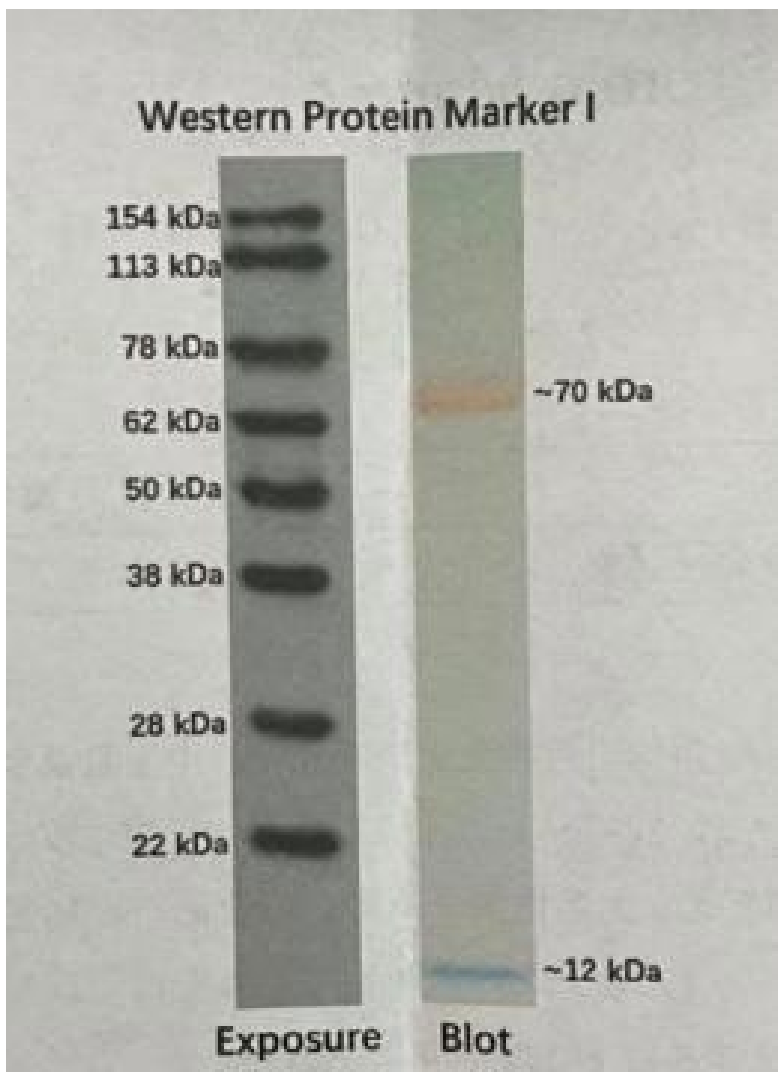

Original Image For Fig 1A

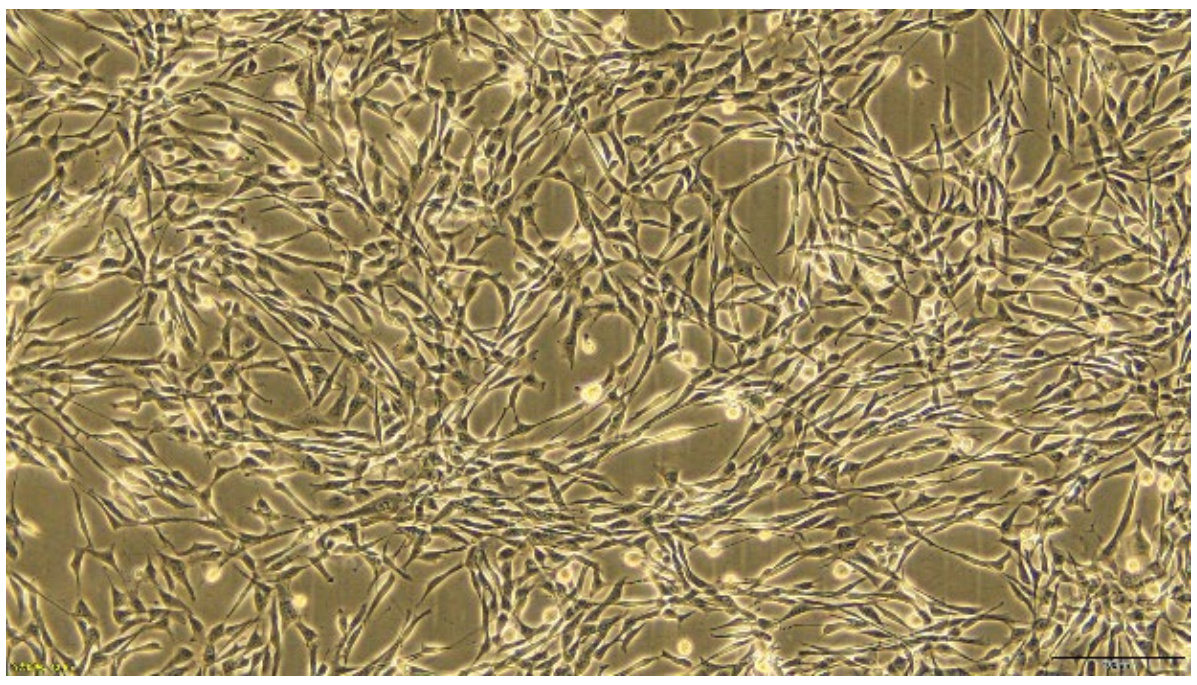

Original Image For Fig 1A

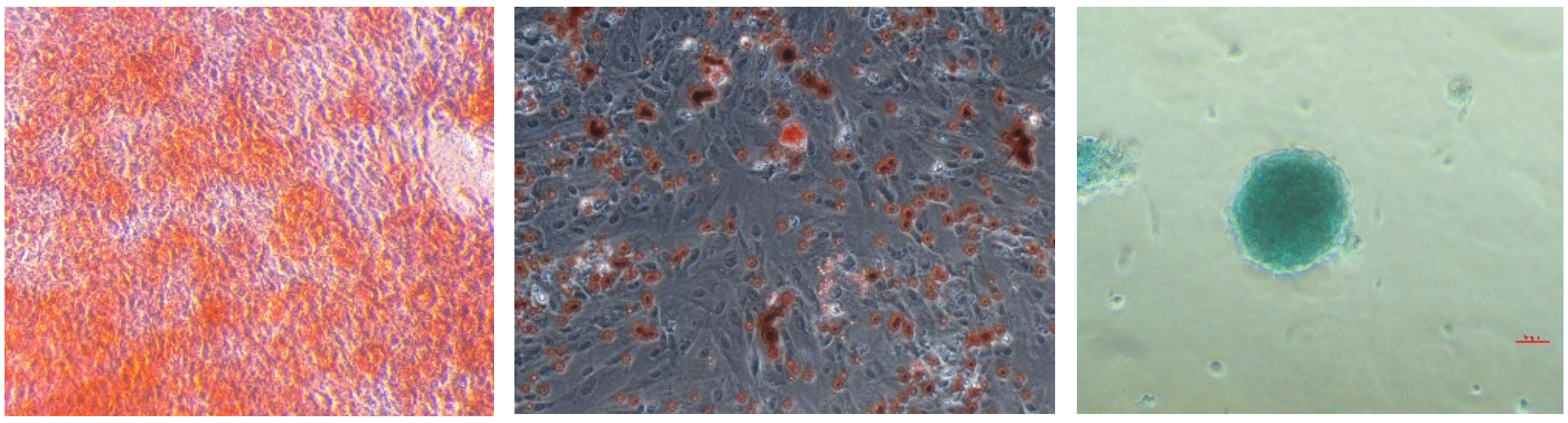

Original Image For Fig 3A

TSG-6

β-Tubulin

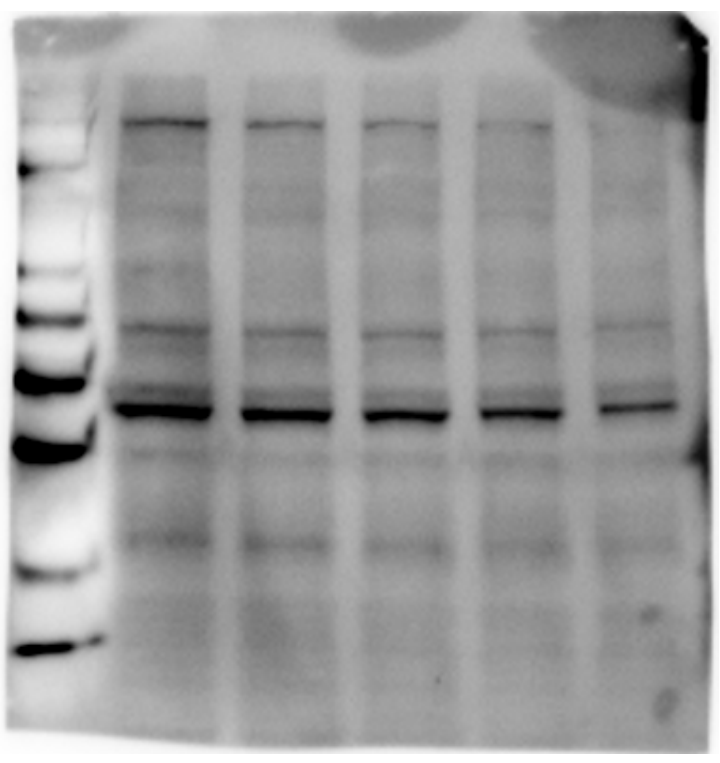

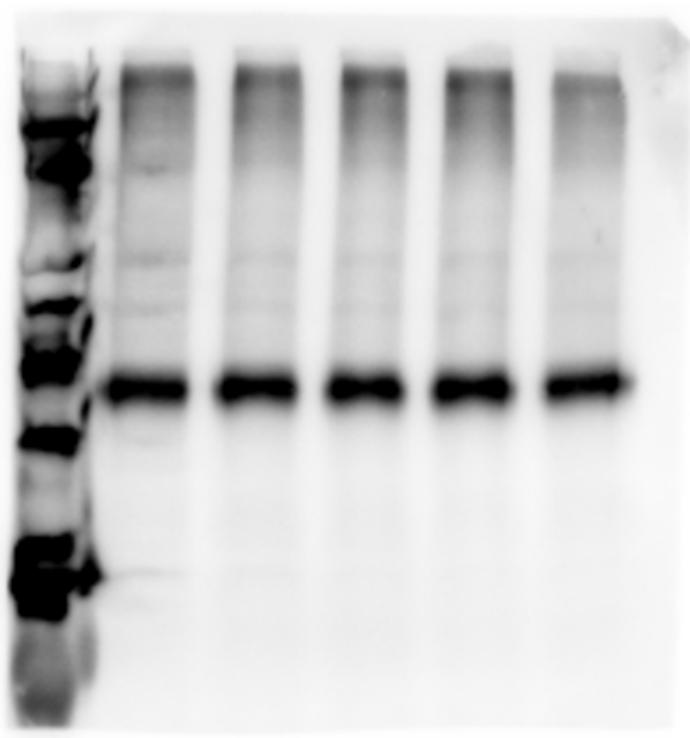

Original Image For Fig 4A

Atg5 LC3 β-Tubulin

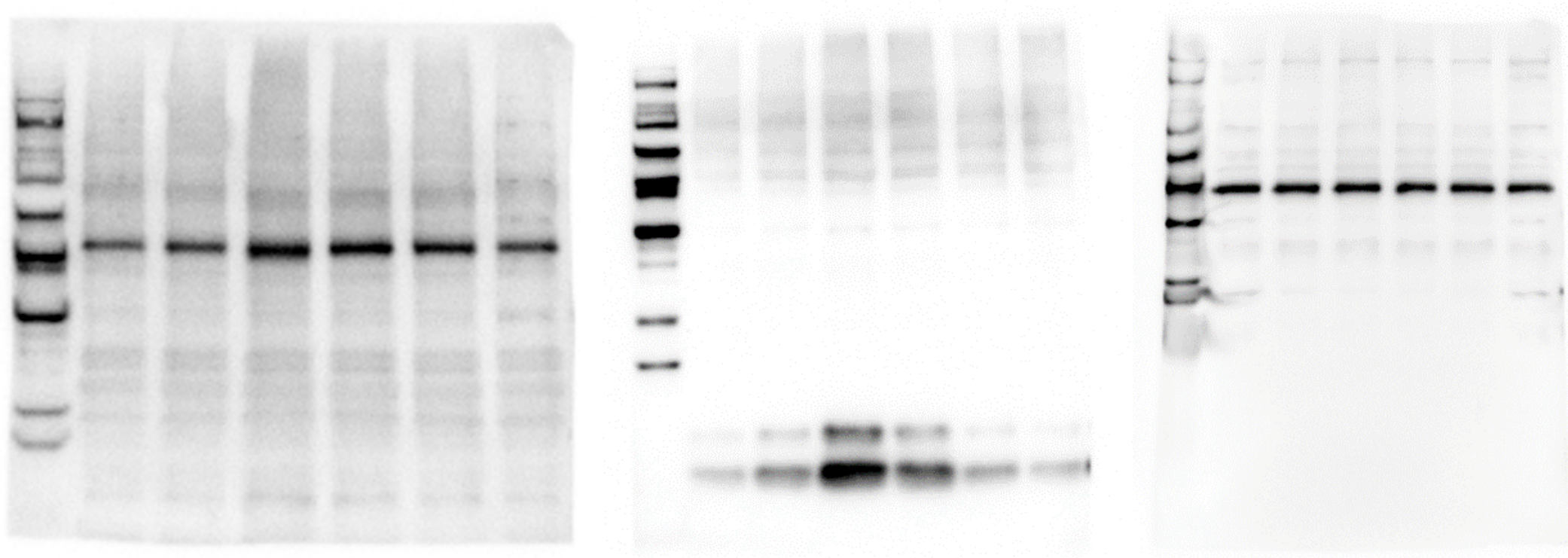

Original Image For Fig 4B

P62 LC3 β-Tubulin

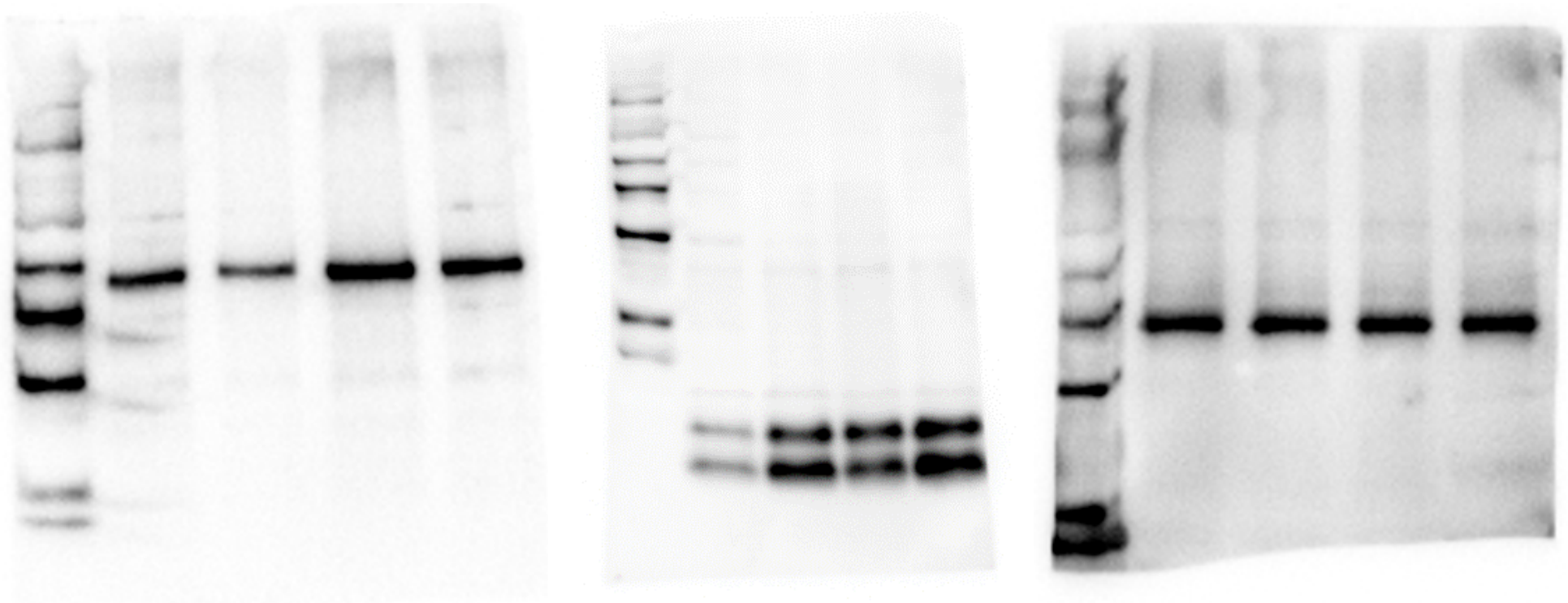

Original Image For Fig 4C

NC

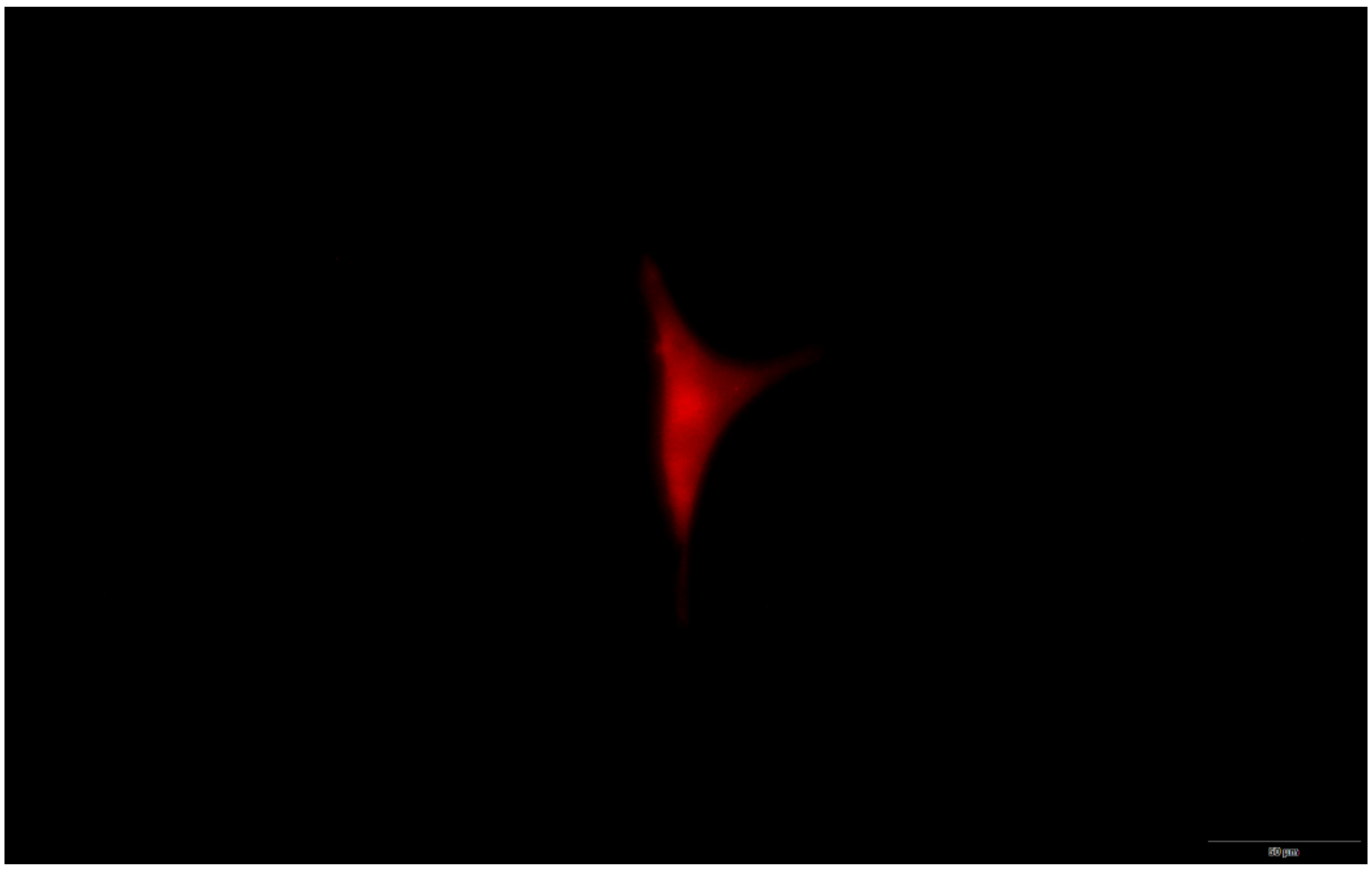

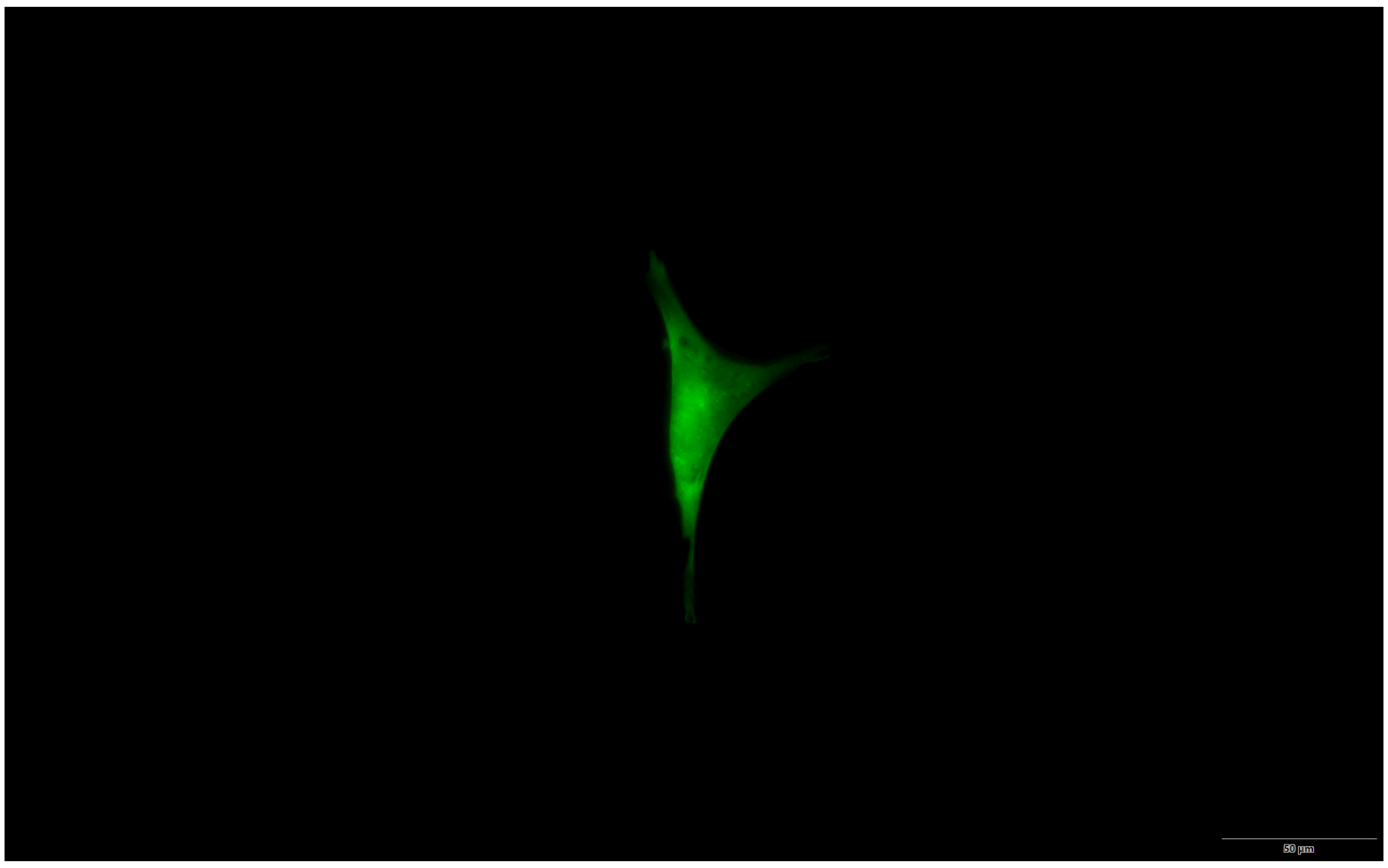
50 µm
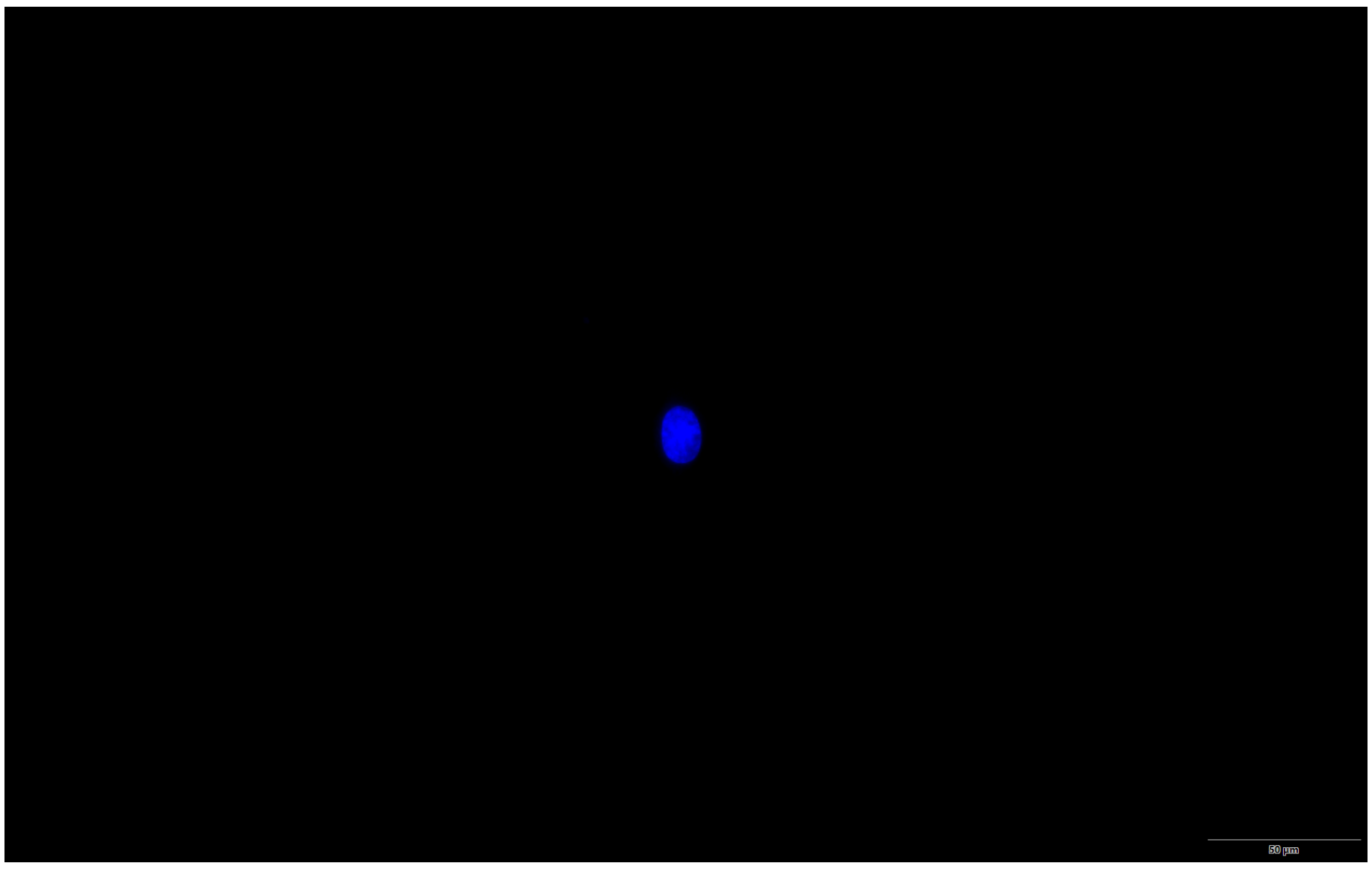
50 µm
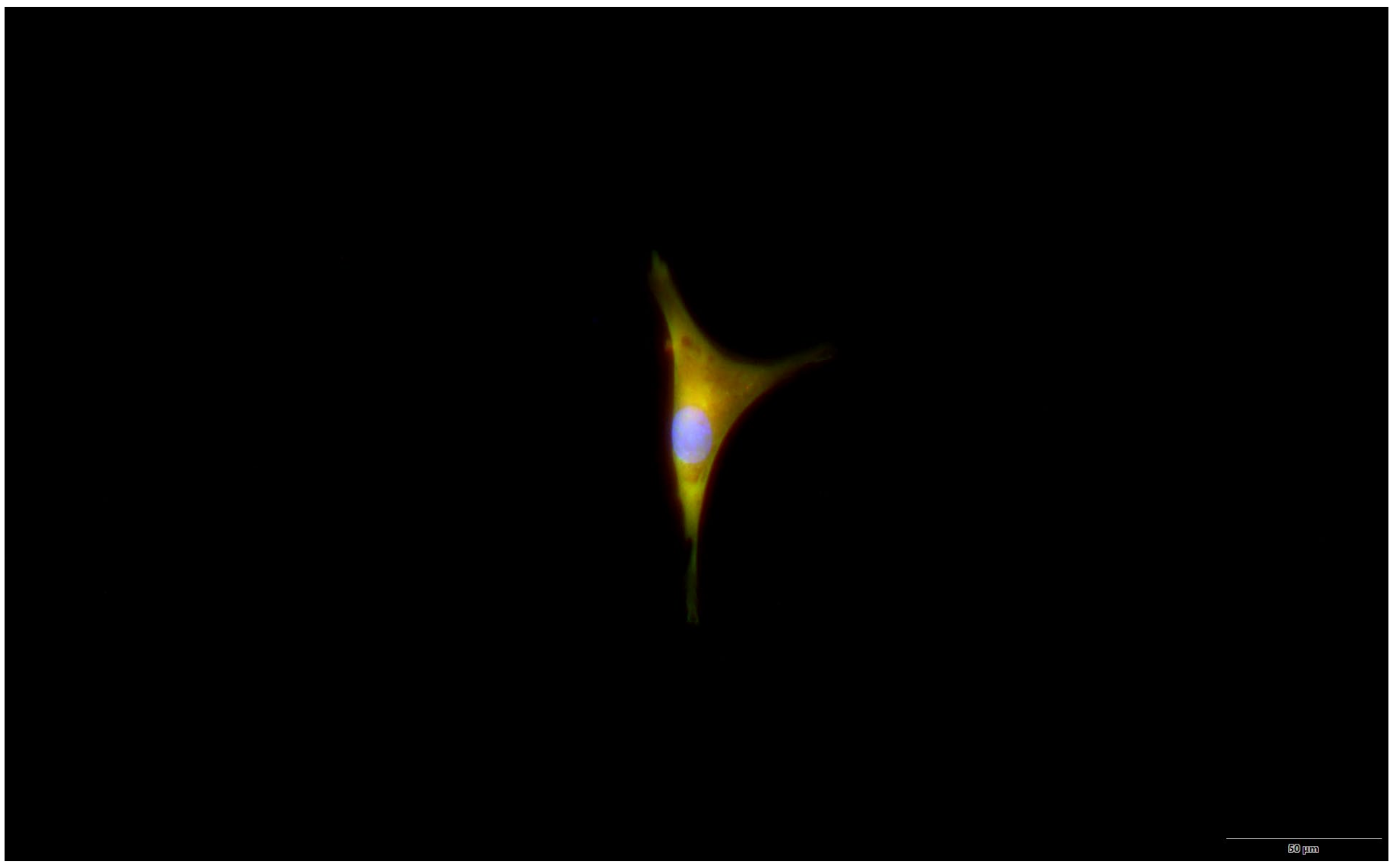
50 µm

Leptin

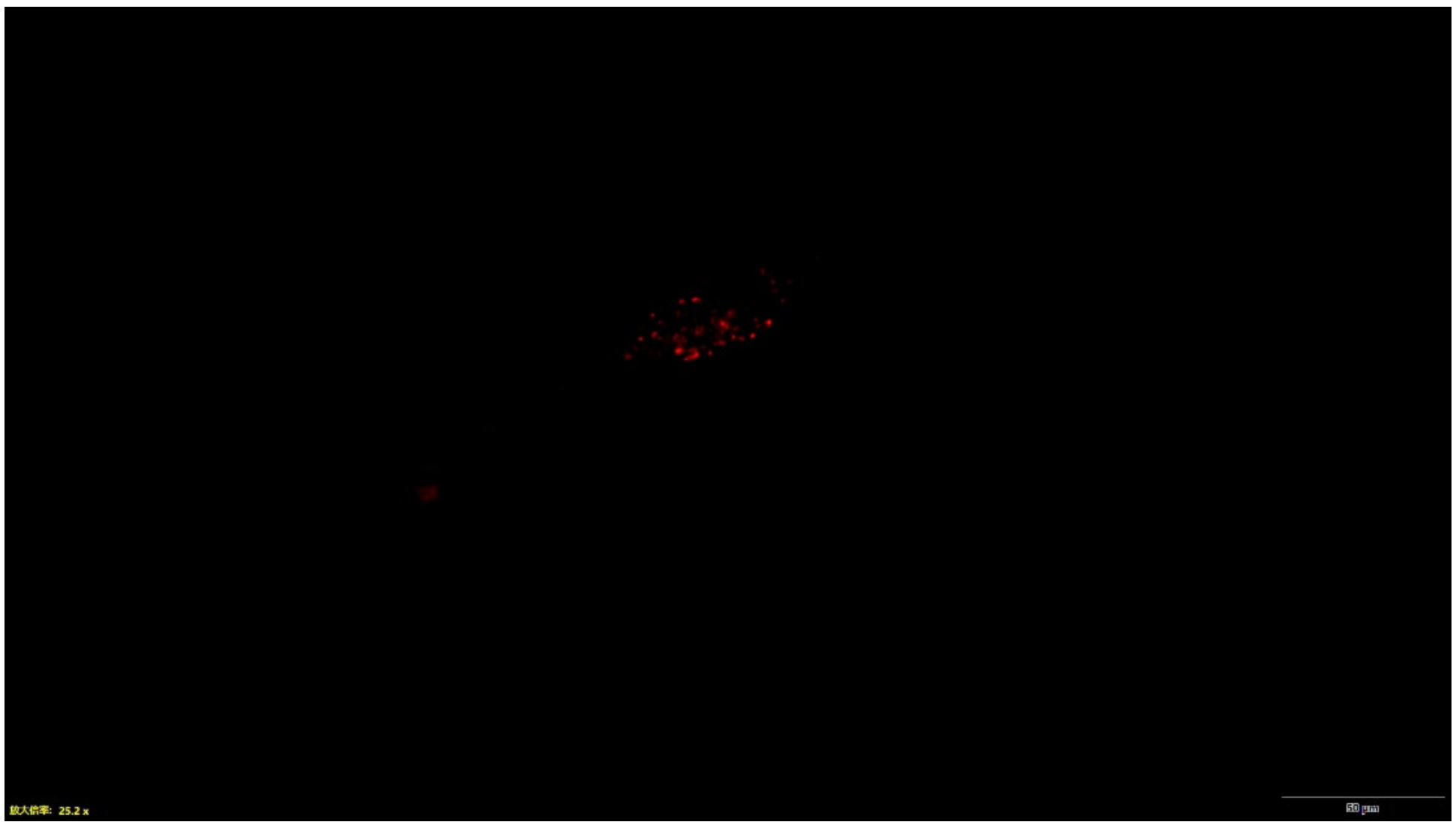

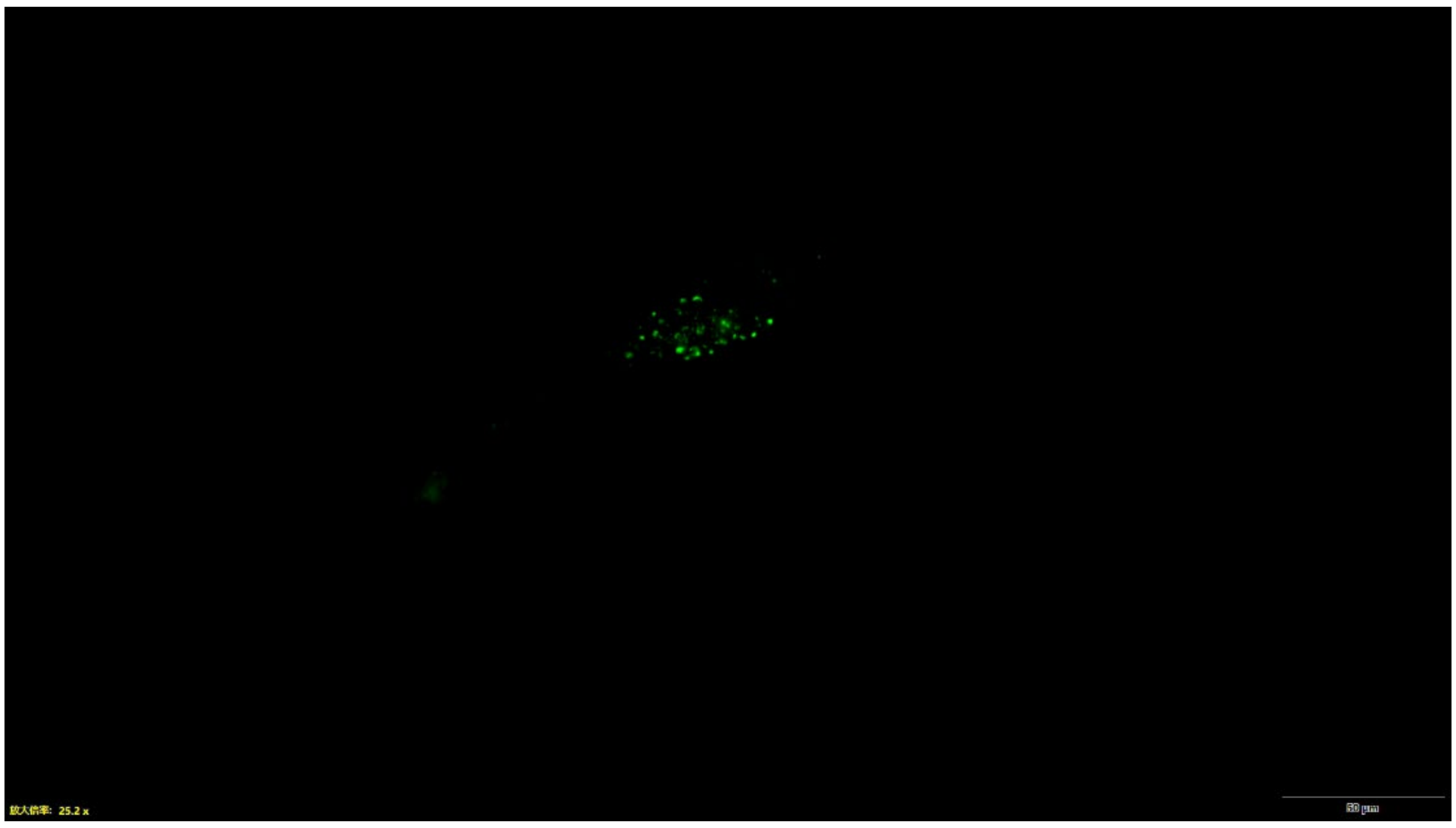

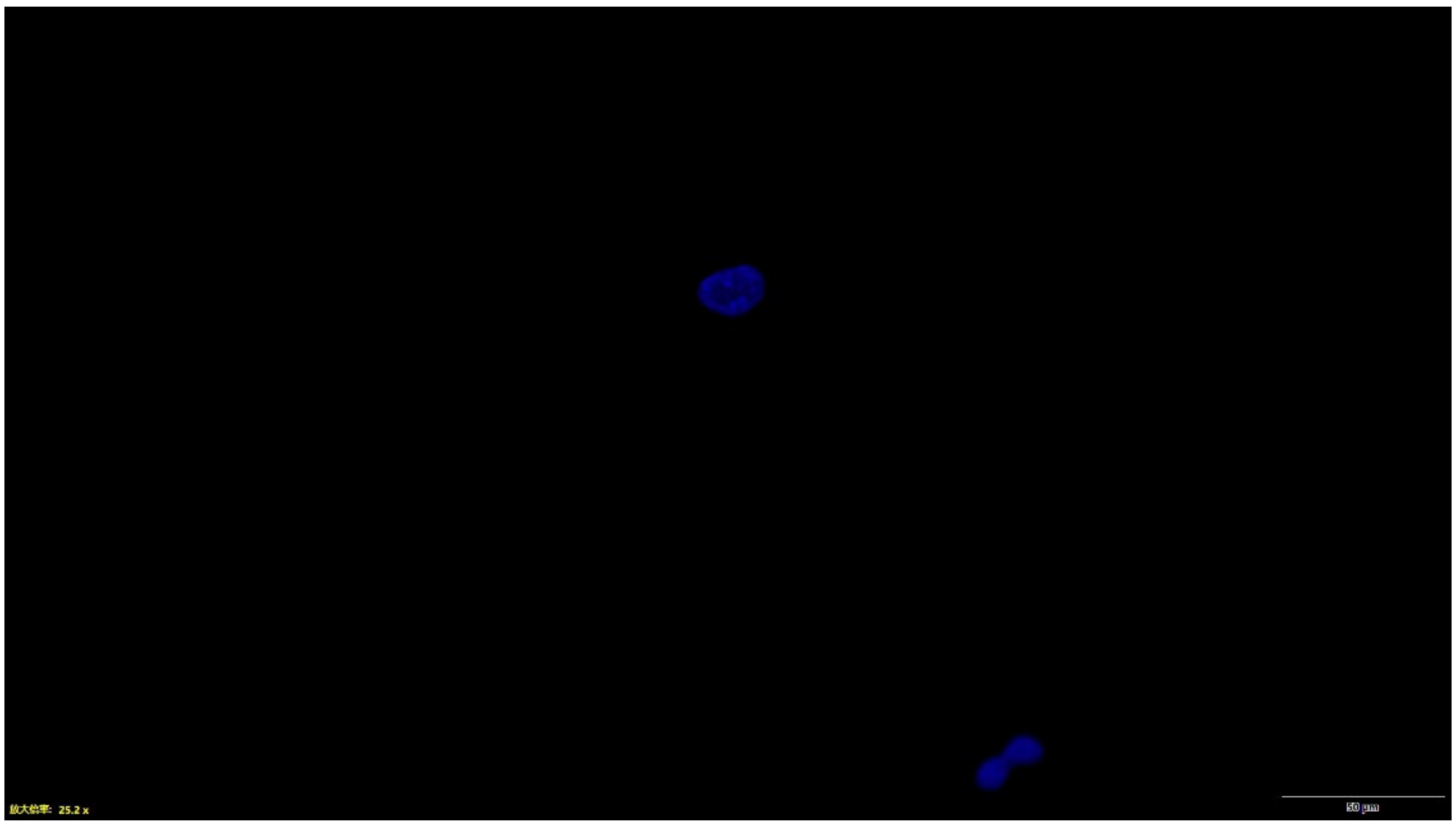

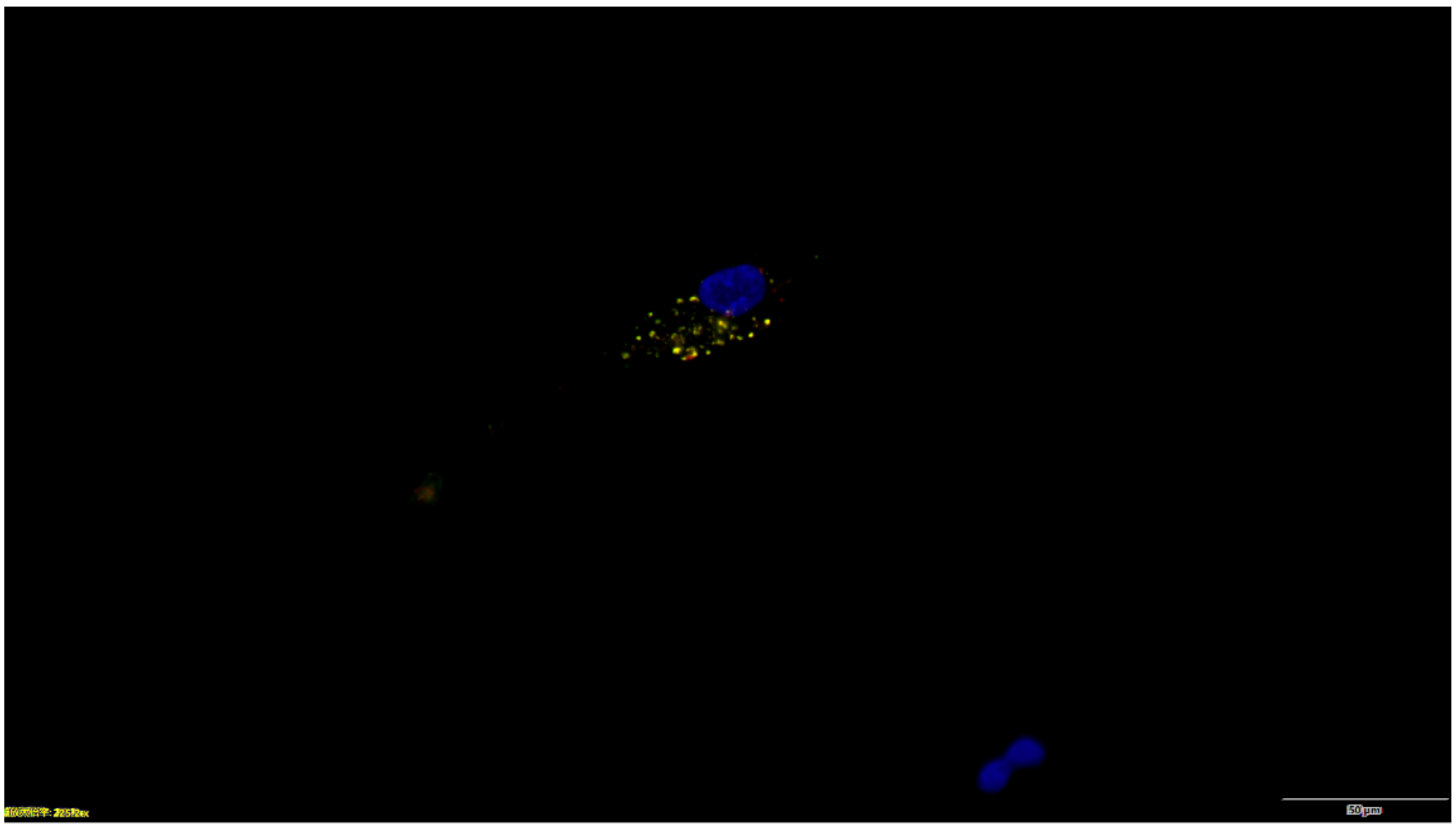

Original Image For Fig 5A

Atg5 LC3 TSG-6 β-Tubulin

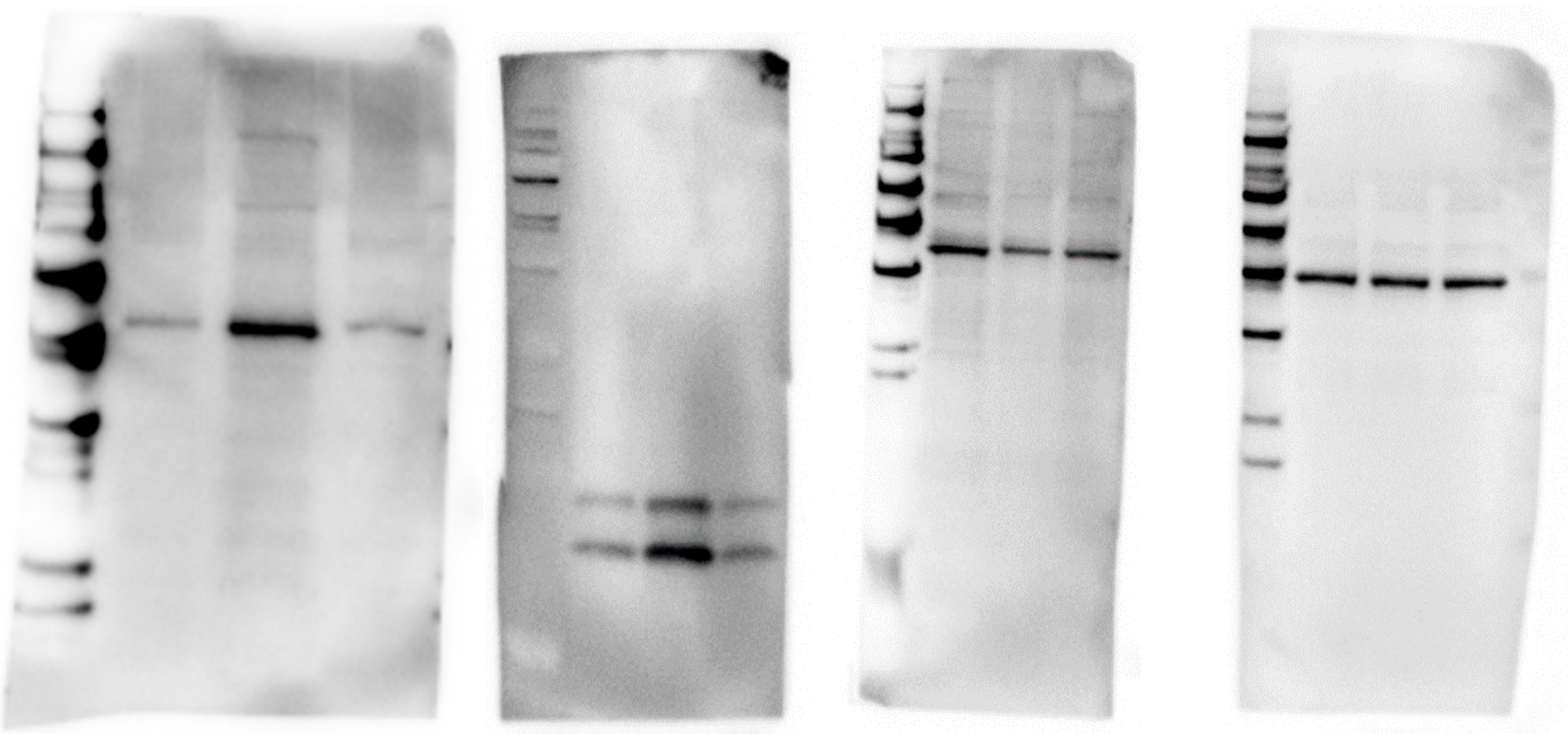

Original Image For Fig 5C

IP: p62 TSG-6

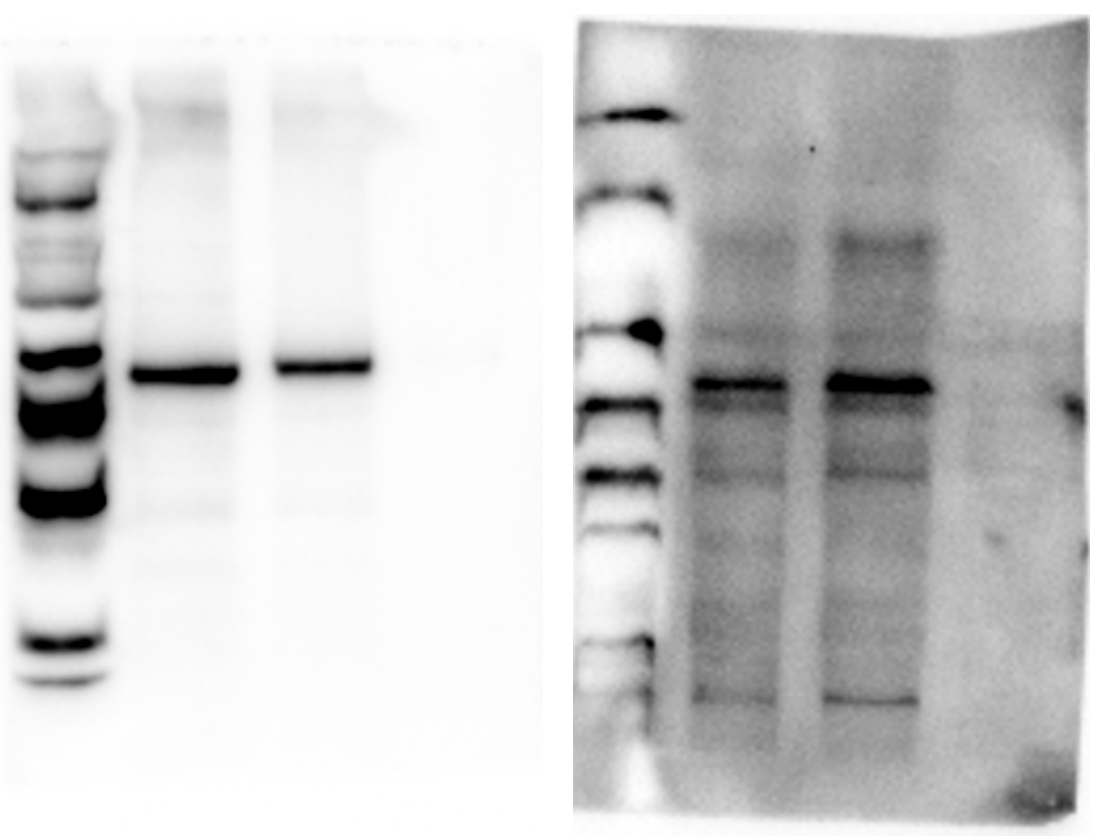

IB: p62 TSG-6 β-Tubulin

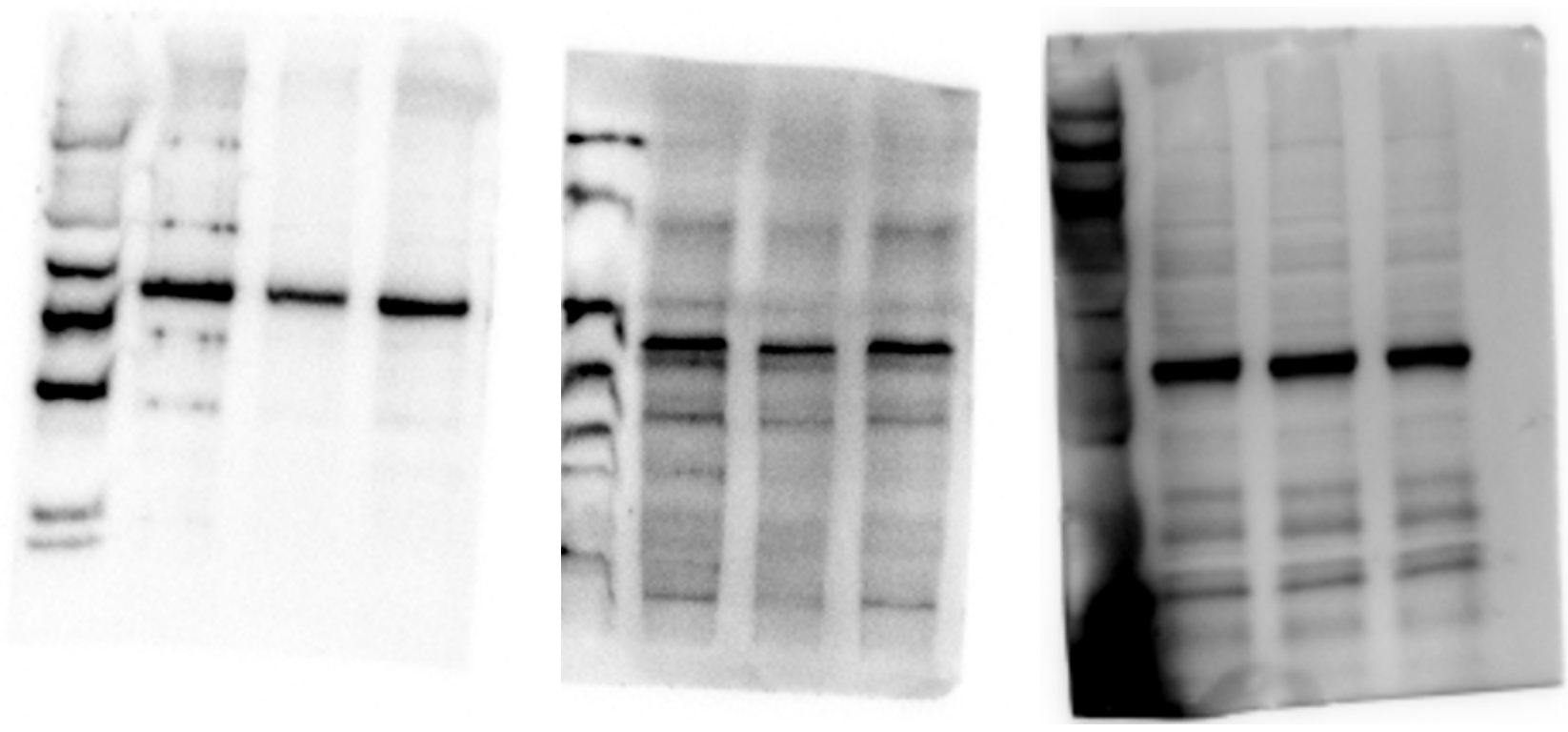

Original Image For Fig7A

Total-p38 p-p38 β-Tubulin

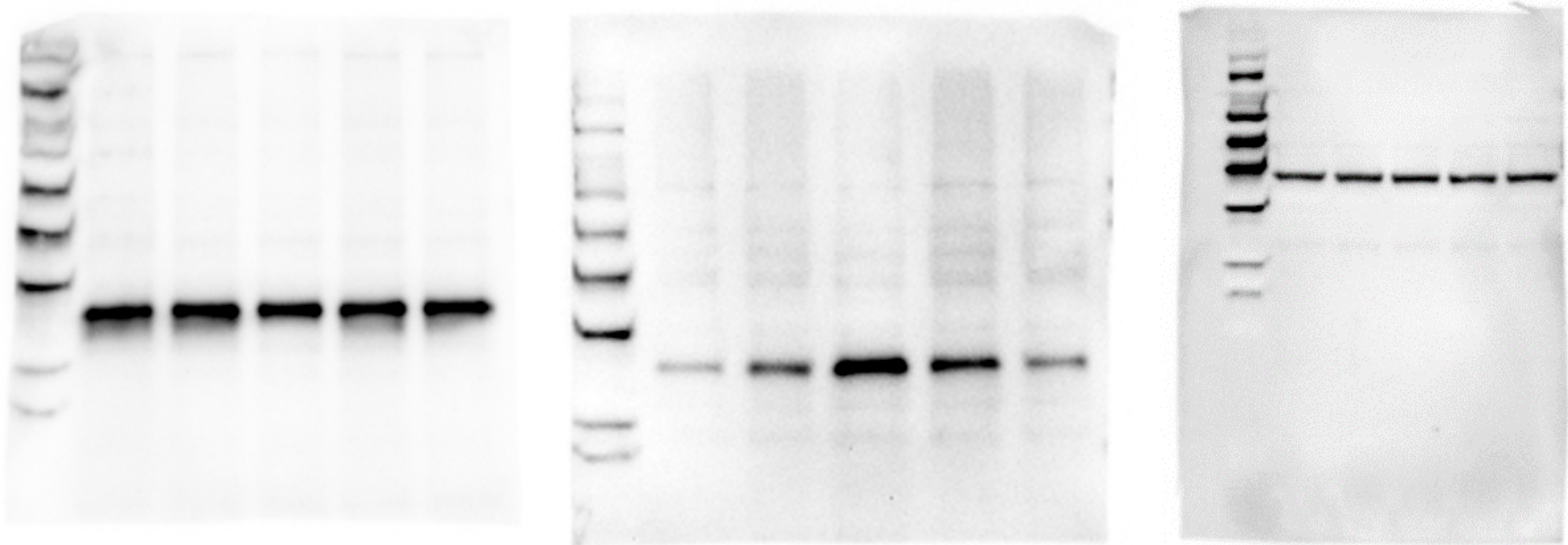

Original Image For Fig7B

p-p38 Atg5 LC3 TSG-6

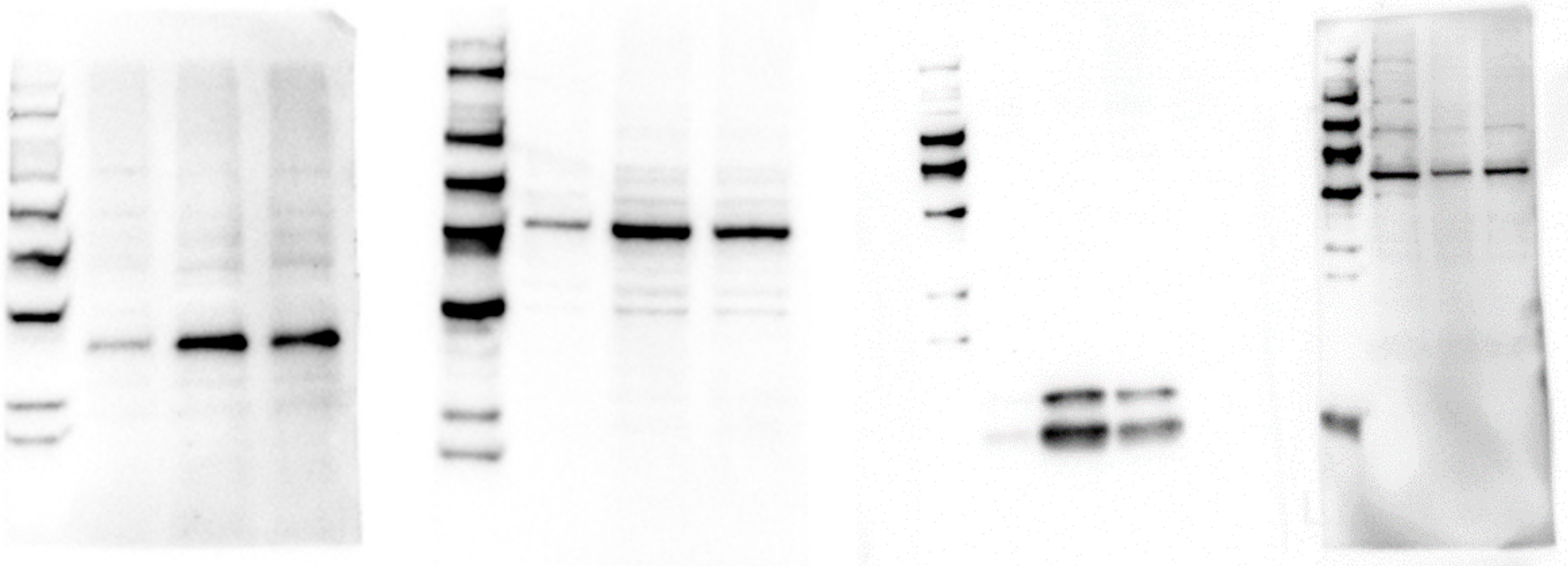

β -Tubulin

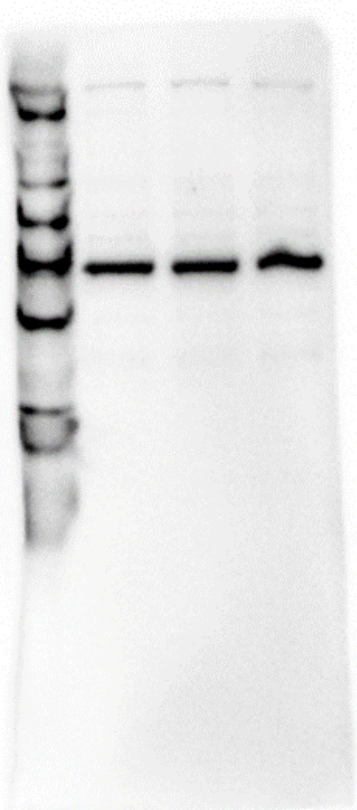

Supplement: Supplementary file 1 [file 41598_2026_57830_MOESM2_ESM.pdf]
