# Supplementary material for: Leptin-induced autophagy regulates the immunomodulatory potential of adipose-derived mesenchymal stem cells through down regulating the expression of TSG-6
Source: Sci Rep. 2026 Jun 15;16:27363. doi: 10.1038/s41598-026-57830-6 (PMC13530140; doi:10.1038/s41598-026-57830-6)

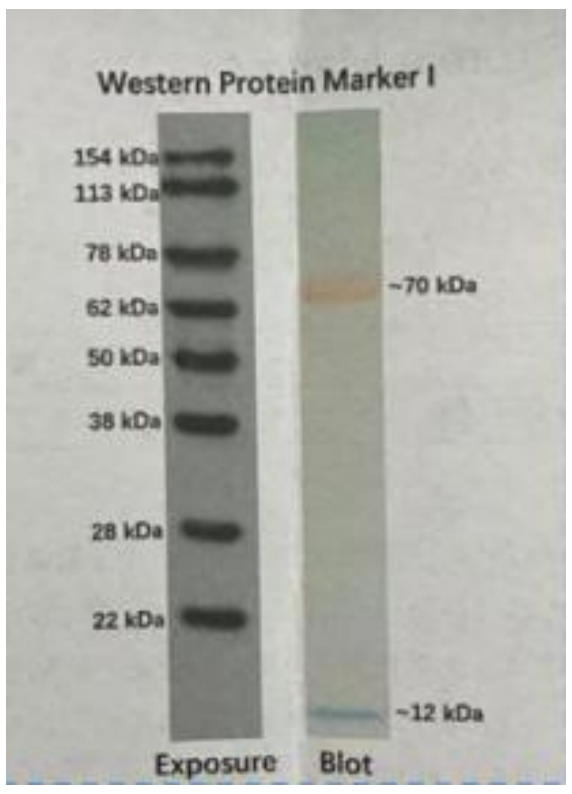

Original Image For S1

TSG6 β-Tubulin

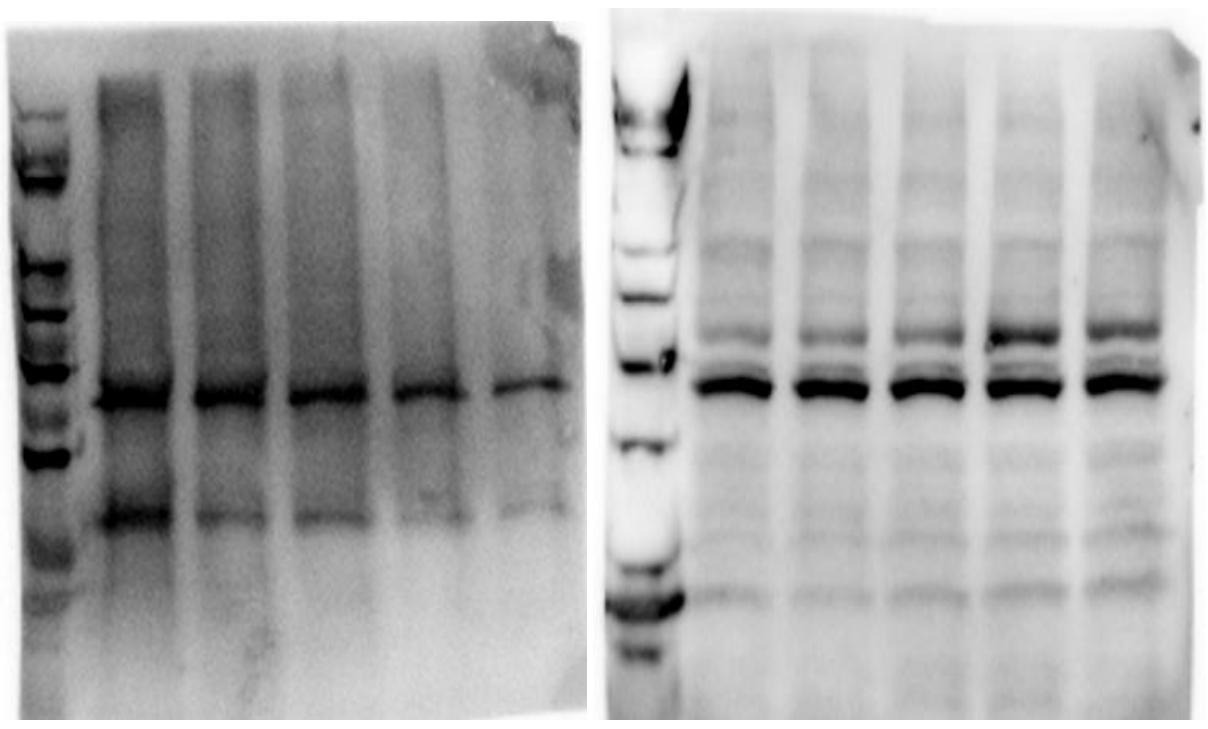

Supplement: Supplementary file 2 [file 41598_2026_57830_MOESM5_ESM.pdf]
