# Supplementary material for: Leptin-induced autophagy regulates the immunomodulatory potential of adipose-derived mesenchymal stem cells through down regulating the expression of TSG-6
Source: Sci Rep. 2026 Jun 15;16:27363. doi: 10.1038/s41598-026-57830-6 (PMC13530140; doi:10.1038/s41598-026-57830-6)
Supplement: Supplementary file 3 [file 41598_2026_57830_MOESM1_ESM.docx]

**Fig. 1.** Characterization of isolated adipose-derived mesenchymal stem cells (ADMSCs) in serum-free medium. (A) The morphology of ADMSCs exhibited the typical spindle shape of MSCs. (B) ADMSCs were highly positive for mesenchymal stem cells surface markers, including CD73, CD90, and CD105, but negative for CD14, CD34, CD45 and HLA-DR by flow cytometry analysis. (C) The multipotency of ADMSCs was detected via osteogenic, adipogenic, and chondrogenic differentiation using alizarin red staining, oil red O staining and alcian blue staining. Representative data from 3 separate experiments are shown. Bars in A and C: 100 µm

**Fig. 2.** The immunosuppressive effect of ADMSCs on macrophage activation were restrained by leptin pretreatment. The LPS-stimulated macrophages were co-cultured with ADMSCs (with or without leptin), and the macrophage-associated markers were detected by flow cytometry (A), and the macrophage-associated cytokines were detected by ELISA (B). Representative data from three separate experiments are shown. **P* < 0.05, ***P* < 0.01.

**Fig. 3.** Leptin downregulated the expression of TSG-6 in ADMSCs. (A) ADMSCs were treated with different concentrations of leptin (0, 50, 100, 250 and 500 ng/mL), and the expressions of TSG-6 were detected by western blot analysis. (B) The effect of leptin on the expression of TSG-6 mRNA was determined by RT-PCR. (C) The effect of leptin on the secretion of TSG-6 in cell culture medium was detected by ELISA. Representative data from three separate experiments are shown. **P* < 0.05, ***P* < 0.01.

**Fig. 4.** Leptin induced autophagy in ADMSCs. (A) ADMSCs were treated with leptin at various periods of time, and the expression of LC3-II was detected by western blot at the indicated times. (B) ADMSCs were pretreated with chloroquine (CQ) and stimulated with leptin, the expressions of p62 and LC3-II were evaluated by western blot analysis. (C) ADMSCs stimulated with or without leptin, the process of autophagic flux was observed by mRFP-GFP-LC3 adenovirus double label assay. Representative data from three separate experiments are shown. **P* < 0.05, ***P* < 0.01.

**Fig. 5.** Leptin-induced autophagy inhibited the expression of TSG-6. ADMSCs were infected with control lentivirus (sh-NC) or lentivirus expressing shRNA targeting Atg5 (sh-Atg5), and treated with or without leptin. The expressions of Atg5, LC3-II and TSG-6 were analyzed by western blot analysis (A). ADMSCs were infected with sh-NC and sh-Atg5 lentivirus and treated with leptin, the expression of TSG-6 protein was determined by ELISA (B). The interaction between p62 and TSG-6 in Leptin-treated ADMSCs was examined via a Co-IP assay (C). Representative data from three separate experiments are shown. **P* < 0.05, ***P* < 0.01.

**Fig. 6.** Autophagy inhibition alleviated leptin’s effects on macrophage polarization. Leptin-stimulated ADMSCs transfected with sh-Atg5 or sh-NC were co-cultured with LPS-stimulated macrophages. The macrophage-associated markers were detected by flow cytometry (A), and the macrophage-associated cytokines were detected by ELISA (B). Representative data from three separate experiments are shown. **P* < 0.05, ***P* < 0.01.

**Fig. 7.** Leptin downregulated the expression of TSG-6 in ADMSCs by autophagy via the activation of p38 MAPK pathway. (A) ADMSCs were treated with leptin, and the expression of MAPK pathway proteins was detected by western blot. (B) ADMSCs were pretreated with p38 MAPK inhibitor SB203580 prior to leptin treatment, and the expressions of p-p38, Atg5，LC3-II, and TSG-6 were assessed by western blot analysis. Representative data from three separate experiments are shown. **P* < 0.05, ***P* < 0.01.
