# Supplementary material for: Leptin-induced autophagy regulates the immunomodulatory potential of adipose-derived mesenchymal stem cells through down regulating the expression of TSG-6
Source: Sci Rep. 2026 Jun 15;16:27363. doi: 10.1038/s41598-026-57830-6 (PMC13530140; doi:10.1038/s41598-026-57830-6)
Supplement: Supplementary file 4 [file 41598_2026_57830_MOESM3_ESM.docx]

Figure S1. Dose‑response effects of leptin on TSG‑6 expression and macrophage polarization.

Western blot analysis of TSG‑6 expression in ADMSCs treated with leptin at 10, 50, 100, and 250 ng/mL(A).Flow cytometric analysis of CD86 expression in LPS‑activated macrophages co‑cultured with ADMSCs pretreated with leptin at 10, 50, 100, or 250 ng/mL(B).Cells in the NC group were left untreated. Data are presented as representative images and plots from three independent experiments.
