# Supplementary material for: Leptin-induced autophagy regulates the immunomodulatory potential of adipose-derived mesenchymal stem cells through down regulating the expression of TSG-6
Source: Sci Rep. 2026 Jun 15;16:27363. doi: 10.1038/s41598-026-57830-6 (PMC13530140; doi:10.1038/s41598-026-57830-6)
Supplement: Supplementary file 5 [file 41598_2026_57830_MOESM4_ESM.docx]

Figure S2. Validation of key findings in human primary macrophages.

Human primary macrophages were left untreated (NC), stimulated with LPS, or co‑cultured with ADMSCs in the absence or presence of leptin (250 ng/mL).CD86 expression was analyzed by flow cytometry. Representative flow cytometry plots from three independent experiments are shown.
